# Dynamical decoration of stabilized-microtubules by Tau-proteins

**DOI:** 10.1038/s41598-019-48790-1

**Published:** 2019-08-28

**Authors:** Jordan Hervy, Dominique J. Bicout

**Affiliations:** 1 Institut Laue-Langevin, 71 Avenue des Martyrs, 38042 Grenoble, France; 2 Laboratory of Physics and Modelling of Condensed Matter, Grenoble Alpes University, CNRS, Grenoble, France; 3 EPSP, TIMC Laboratory, UMR CNRS 5525 Grenoble Alpes University, VetAgro Sup, Grenoble, France

**Keywords:** Computational biophysics, Computational models

## Abstract

Tau is a microtubule-associated protein that regulates axonal transport, stabilizes and spatially organizes microtubules in parallel networks. The Tau-microtubule pair is crucial for maintaining the architecture and integrity of axons. Therefore, it is essential to understand how these two entities interact to ensure and modulate the normal axonal functions. Based on evidence from several published experiments, we have developed a two-dimensional model that describes the interaction between a population of Tau proteins and a stabilized microtubule at the scale of the tubulin dimers (binding sites) as an adsorption-desorption dynamical process in which Tau can bind on the microtubule outer surface via two distinct modes: a longitudinal (along a protofilament) and lateral (across adjacent protofilaments) modes. Such a process yields a dynamical distribution of Tau molecules on the microtubule surface referred to as *microtubule decoration* that we have characterized at the equilibrium using two observables: the total microtubule surface coverage with Tau’s and the distribution of nearest neighbors Tau’s. Using both analytical and numerical approaches, we have derived expressions and computed these observables as a function of key parameters controlling the binding reaction: the stoichiometries of the Taus in the two binding modes, the associated dissociation constants and the ratio of the Tau concentration to that of microtubule tubulin dimers.

## Introduction

Microtubules are one of the three types of filamentous polymers that constitute the cellular cytoskeleton. A key feature of microtubules is their dynamic nature^1,2^. This dynamical behaviour, referred to as *dynamic instability*, is exquisitely regulated and is crucial to many cellular activities including cell division, intracellular transport and the establishment and maintenance of cell shape and polarity^3^. Tau (Tubulin Associated Unit) is an important microtubule-regulating protein that is predominantly expressed in axons^4^. This neuronal protein has been reported to cover a large range of fundamental microtubule-related functions. In particular, Tau promotes tubulin assembly^5,6^, stabilizes (i.e, regulates) the dynamic instability of microtubules^7,8^, spatially organizes microtubules in a parallel network in axons^9^ and can control the axonal transport in regulating the walk of kinesins and dyneins along microtubules^10^. Overall, Tau significantly contributes to the stabilization of neuronal microtubules, although the mechanisms underlying these biological functions are still not well understood. Furthermore, appearance of dysfunctions in the couple Tau-microtubule has been correlated with numerous neurodegenerative diseases commonly referred as *Tauopathies* including Alzheimer’s, Huntington’s and Pick’s diseases^11–13^. This group of neurodegenerative diseases is characterized by an accumulation of abnormal Tau protein in the human brain^14^. Both gain of toxicity and loss of normal function of Tau-proteins are though to contribute to the development of *Tauopathies*^3,15^.

**Figure 1:**
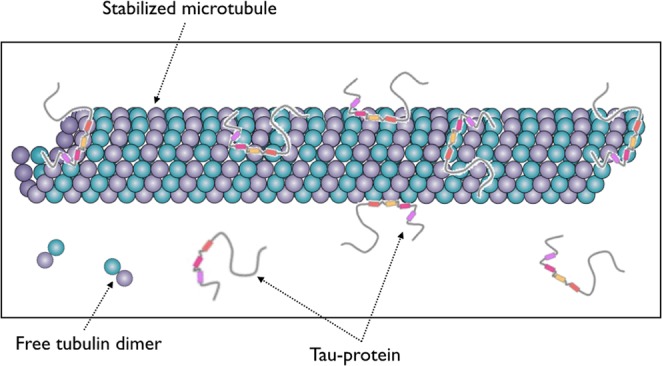
Cartoon showing a population of Tau’s binding on/unbinding from a stabilized microtubule. Adapted from^56,57^.

Because of its important implication in neurodegenerative disorders, Tau has been the focus of much study, with a recent emphasis on Tau-based therapeutic strategies^16,17^. To understand how Tau ensure the essential normal functions, it is of paramount importance to figure out how it interacts with microtubules. In addition to the long-standing experimental effort, simulations of the molecular dynamics of the Tau protein along with a MT section have recently been performed^18^. In this study, we are interested in modeling the reversible binding reaction between a population of Tau-proteins and stabilized-microtubules. As illustrated in Fig. 1, for a given concentration of Tau in the solution and a given concentration of polymerized tubulin dimers forming the microtubule, the binding reaction yields to a dynamical distribution of Tau on the microtubule surface, which we will refer to as *microtubule decoration*. The main objective of this article is to develop a modeling framework for describing the decoration of microtubules using the average number of bound Tau and the spatial distribution of Tau on the surface of microtubules. To this end, we have developed a general decoration model based on data from published literature. For this purpose, two aspects have been taken into account. First is the description of the lattice structure of the microtubule surface forming the playground where Tau proteins bind. Second, the characteristics of how Tau proteins interact specifically with a stabilized microtubule, including the structure of Tau protein, the definition and location of Tau binding sites on the microtubule, and the parameters of the binding reaction. A state-of-the-art knowledge database on these aspects has been constructed from the literature data (see the Supplementary Information, Sec. S3) and the main results summarized below.

## Summary of the Literature Analysis

### Microtubule lattice

**Figure 2:**
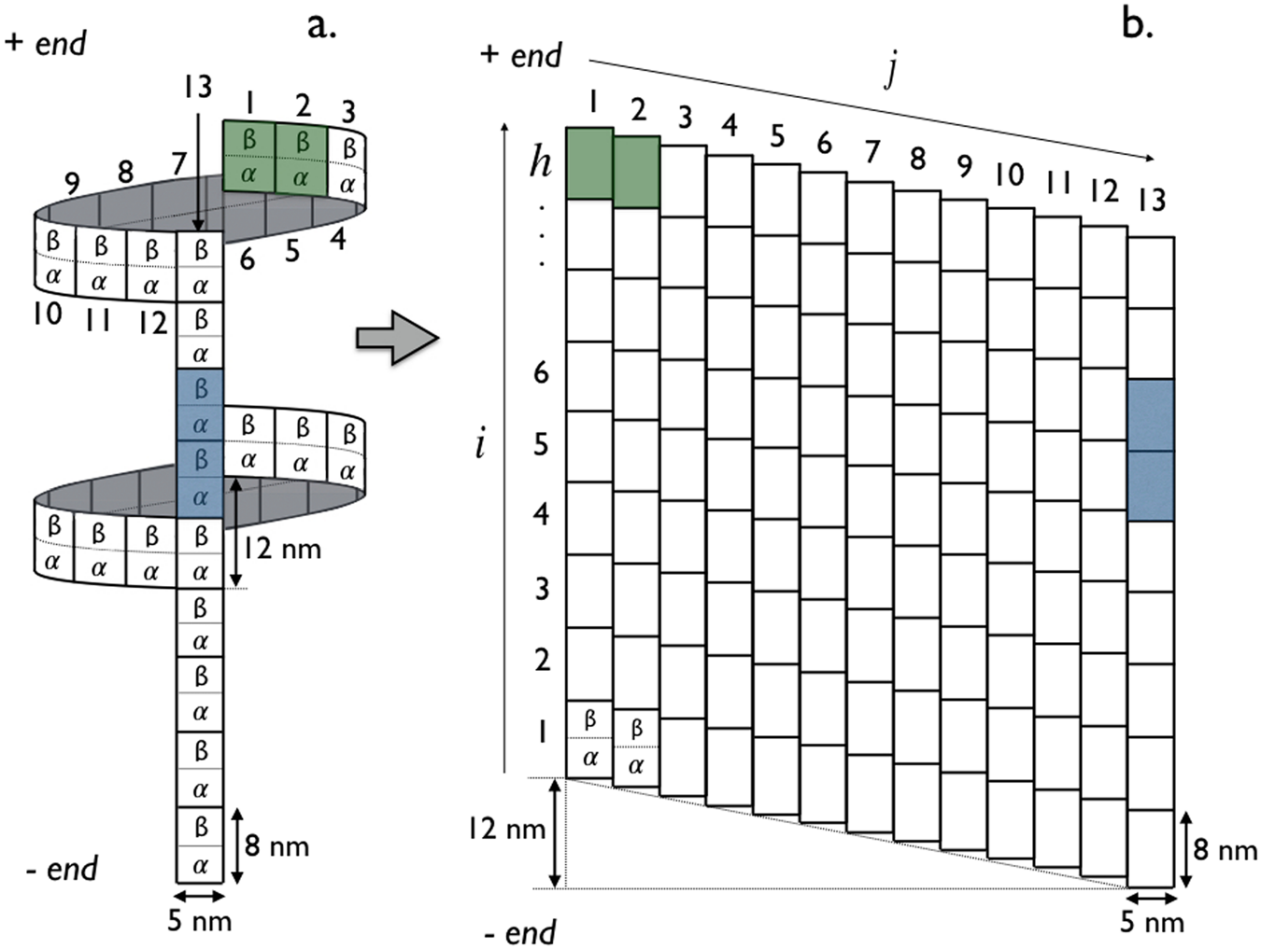
Tau’s binding sites on the microtubule representations (see main text for details). Modes “*p*” (blue) and “*h*” (green) of binding are illustrated in dark color. (**a**) Three-dimensional representation of the 13–protofilament microtubule. (**b**) Two-dimensional lattice mapping of the 13–protofilament microtubule with *N* = *h* × *p* lattice sites (tubulin dimers) where *h* is the number of *αβ*-tubulin dimers along the protofilament axis *i* and *p* = 13 is the number of protofilaments (or *αβ*-tubulin dimers along the helix axis *j*). Adapted from the manuscript of J. H.’s thesis^58^.

Microtubules (MT) are composed of 8 *nm* long *αβ*-tubulin dimers which are aligned end-to-end to form linear protofilaments^19^. Most of the microtubules assembled *in vitro* and *in vivo* are composed of *p* = 13 protofilaments^20^ with a longitudinal shift of 12/13 ≈ 0.92 *nm* between protofilaments, generating a left-handed three-start helix^21–23^. As shown in Fig. 2a,b, the distance separating two protofilaments is about 5 *nm*^21^. At the microscopic level, *αβ*-tubulin heterodimers are packed in a B-type lattice, which has been found to be the most favorable configuration^24^. Interactions between protofilaments in the lattice involve homologous subunits (*α* – *α* and *β* – *β*) except at the seam (i.e., between the first and last protofilament), where a discontinuity exists due to the pitch of three tubulin monomers. The microtubule is a polar structure with an “+end” extremity exhibiting *β* monomers and an “−end” extremity exhibiting *α* monomers. In this study, we assumed that the microtubule curvature because of its helical geometry has no effect on the binding of Tau molecules and that the 13-protofilaments consisting the microtubule are all identical.Therefore, we use the unfold and flattened bi-dimensional lattice representation shown in Fig. 2b as an appropriate model of the MT surface for the Tau-protein binding process.

### Tau-microtubule interactions

Four items are to be considered for the purpose of this work.

**Figure 3:**
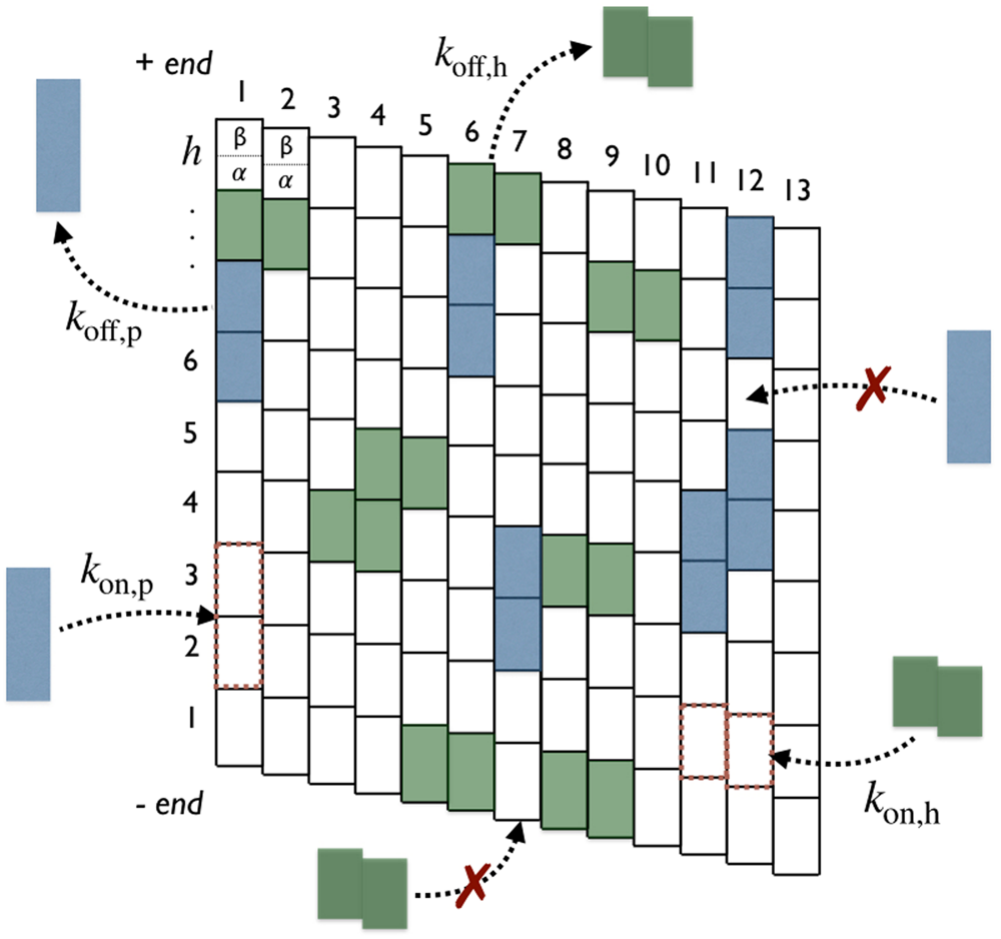
Binding rules on the microtubule lattice (*N* = 9 × 13 sites). Attachment and detachment of Tau’s in modes “*p*” (blue) and “*h*” (green) are represented by incoming and outgoing arrows, respectively, with respective rates: *k*_on,p_, *k*_off,p_, *k*_on,p_ and *k*_off,h_. The on-rate is conditional to free available binding sites. Forbidden attachments are indicated by red crosses. Adapted from the manuscript of J. H.’s thesis^58^.
- Tau binding stoichiometry (Tau:binding site ratio): Most of the values reported in the literature seem to converge towards stoichiometry of 0.5; *ν* = 0.4^39–41^, *ν* = 0.412^42^, *ν* = 0.46^43^ and *ν* = 0.52^31^. In addition, when the above-mentioned stoichiometries are corrected following the approach in^44^, one ends up with *ν* = 0.5 corresponding to “1” Tau molecule for “2” *α* − *β*-tubulin dimers (i.e., 1 Tau for 4 tubulin monomers). These experimental evidences are supported by molecular dynamics simulations showing that bound Tau molecules are in an extended conformation and interact with two *α* − *β*-tubulin dimers on average^18^. However, a recent study suggests that Tau can as well span up to 4 *α* − *β*-tubulin dimers leading to a smaller stoichiometry of *ν* = 0.25^32^. Therefore, to keep generality in this work, the model of decoration presented below will be general to allow to consider any stoichiometry. However, the case of *ν* = 1/2 will often be used in illustrations.

- Tau protein structure: Tau is a “natively unfolded” molecule with a radius of gyration of 5–7 *nm* in solution^19,25^. There are 6 variants of the Tau-protein called isoforms which are distinguished by their amino acid sequences. Tau can be regarded as a dipole with two domains of opposite charge, a microtubule-binding domain involving 3 (Tau 3R) or 4 (Tau 4R) sequence repeats and a projection domain regulating the spacing between microtubules in axons^26,27^. It has been shown that repeats bind independently of each other and that the binding affinity increases with the number of repeats^28^.
- Tau-MT binding sites: The exact Tau-MT binding sites are still not well defined^29^. Comparisons between Tau decorated and control microtubules using cryo-electron microscopy revealed that binding of Tau proteins occurs on the outer surface of microtubules^30–32^; an evidence also supported using atomic force microscopy^33^. On the other hand, a study in 2003 reported a possible binding site on the inner surface of microtubules close to the taxol-binding site on *β*-tubulin^34^. Later on, Makrides *et al*.^35^ suggested that these discrepancies may come from differences in the experimental protocol when adding Tau proteins into the solution with either an addition to pre-stabilized MTs or during polymerizing tubulin. However, a recent high resolution cryo-EM study has shown that in both experimental conditions, Tau was always bound on the outer surface of microtubules^32^. Moreover, the authors in^32^ proposed a model in which Tau interacts with both *α*- and *β*-tubulin. In this study, we will consider that binding of Tau occurs on the outer MT surface with the *α* − *β*-tubulin dimer as the elementary unit of binding site as shown in Fig. 2.
- Tau binding modes: The binding mode and the geometry of Tau when bound to the MT surface is still very controversial. Some studies^31,32,36,37^ have suggested that Tau-proteins preferentially adopt an ordered structure aligning along protofilament ridges when bound on the MT, while structures of bound Tau crossing adjacent protofilaments were observed as well in^33^. And a combination of high-resolution metal-shadowing and cryo-EM has revealed the existence of both longitudinal (along protofilaments) and lateral (across protofilaments) bound Taus on the same MT^30^. This latter observation is consistent with a recent study showing that Tau promotes the formation of tubulin rings alone and stacks of tubulin rings^38^. In the absence of any further information and to keep generality, we will consider in this study that a Tau-protein is likely to bind on the outer MT surface with two binding modes: a longitudinal mode (“*p*” mode), in which the binding occurs along a single protofilament and, a lateral mode (“*h*” mode), where the binding takes place across adjacent protofilaments along the helix, see Figs 2 and 3.

## Results

### Formulation of the decoration model

The model of decoration that we will develop below aims at least to account for all the aspects of the Tau-MT interaction summarized in the Sec. 2.

We consider the problem of reversible binding reaction, as illustrated in Fig. 1, in a system of constant volume with non-interacting Tau-molecules (ligands) at concentration [*Tau*] and identical stabilized and non-dynamic microtubules (macromolecules) at concentration [*MT*]. As depicted in Fig. 2, each microtubule is described as a two-dimensional lattice consisting of *N* = *h* × *p* binding sites (*α* − *β*-tubulin dimers) where *h* and *p* are the number of helices and protofilaments, respectively; the MT lattice representing the outer surface of the MT. A Tau-molecule is visualized as a stem of zero extension and length or size *σ*_*p*_ and *σ*_*h*_ (positive integers) when bound in modes “*p*” and “*h*”, respectively. In this respect, a Tau-molecule bound in the mode “*p*” covers (1 + *σ*_*p*_) consecutive binding sites along a single protofilament (across (1 + *σ*_*p*_) consecutive helices) while it covers (1 + *σ*_*h*_) consecutive binding sites along a single helix (across (1 + *σ*_*h*_) adjacent protofilaments) when bound in the mode “*h*”. The binding of a Tau can only occurs on free lattice binding sites; neither partial, nor overlapping, nor stacked bindings are allowed and no “*h*” mode binding at the seam (i.e., crossing protofilaments *j* = 1 and *j* = 13) is allowed. The binding of Tau is a saturable process; accumulation on the MT surface is not possible. The two-dimensional stoichiometry (Tau:binding sites ratio) matrix associated with these rules writes as,

$$\nu \equiv (\begin{array}{ll}{\nu }_{pp} & {\nu }_{ph}\\ {\nu }_{hp} & {\nu }_{hh}\end{array})=(\begin{array}{ll}\frac{1}{1+{\sigma }_{p}} & 1\\ 1 & \frac{1}{1+{\sigma }_{h}}\end{array}),$$

where diagonal elements *ν*_*pp*_ and *ν*_*hh*_ are the stoichiometries of Taus bound in “*p*” and “*h*” modes, respectively, and the off-diagonal element *ν*_*ph*_ (*ν*_*hp*_) represents the apparent projection stoichiometry along the helix (protofilament) axis for a Tau bound in “*p*” (“*h*”) mode. These binding rules are illustrated and summarized in Fig. 3 for a microtubule lattice of *N* = 9 × 13 binding sites in the case of *ν* = 1/2 corresponding to *σ*_*p*_ = *σ*_*h*_ = 1.

Let *ρ*_*p*_ and *ρ*_*h*_ denotes the coverages (= [concentration of bound Taus]/[concentration of binding sites]) of Taus bound in modes “*p*” and “*h*”, respectively, at any time *t*. In the mean field approximation, the time evolution of *ρ*_*p*_ and *ρ*_*h*_ can be described by the system of coupled non-linear differential equations:

1 $$\{\begin{array}{c}\,\frac{d{\rho }_{p}}{dt}={k}_{\mathrm{on},p}N[MT]\times (x-{\rho }_{p}-{\rho }_{h})\times {p}_{{\rm{binding}},{\rm{p}}}(t|{\rho }_{p},{\rho }_{h})-{k}_{\mathrm{off},p}\,{\rho }_{p},\\ \,\frac{d{\rho }_{h}}{dt}={k}_{\mathrm{on},h}N[MT]\times (x-{\rho }_{p}-{\rho }_{h})\times {p}_{\mathrm{binding},h}(t|{\rho }_{p},{\rho }_{h})-{k}_{\mathrm{off},h}{\rho }_{h},\end{array}$$

where *x* = [*Tau*]/(*N* × [*MT*]) is the Tau:tubulin-dimer ratio of the system. The first positive terms in Eq. (1) describe the increase of the coverages in which a free Tau binds in mode “*i*” (*i* = *p*, *h*) with the rate, *k*_on,i_ × *N* × [*MT*] × *p*_binding,i_(*t*|conf) (per unit of time), on the MT lattice by covering 1 + *σ*_*i*_ consecutive binding sites, where *p*_binding,i_(*t*|conf) is the time and configuration (*ρ*_*p*_, *ρ*_*h*_) dependent probability for a Tau binding in mode “*i*” on the MT lattice (Fig. 3). And, the last negative terms in Eq. (1) describe the decrease of the coverages in which an already bound Tau in mode “*i*” comes off with the rate *k*_off,i_ (per unit of time) leaving unoccupied 1 + *σ*_*i*_ consecutive binding sites (Fig. 3). The coupling between the two sub-lattice p and h in Eq. (1) is mainly ensured by *p*_binding,i_(*t*|conf).

Let’s focus now on the equilibrium situation and define by *ρ*_*p*,*eq*_ and *ρ*_*h*,*eq*_ the equilibrium coverages. For notational simplicity, we will drop in what follows the index “*eq*” on coverages. Thus, the equilibrium densities *ρ*_*p*_ and *ρ*_*h*_ are obtained by setting *dρ*_*p*_/*dt* = *dρ*_*h*_/*dt* = 0 in Eq. (1) and solving the system of equations:

2 $$\{\begin{array}{c}\frac{{\rho }_{p}}{(x-{\rho }_{p}-{\rho }_{h})}={k}_{\mathrm{eq},p}{{\rm{\Phi }}}_{p}({\rho }_{p},{\rho }_{h});\,{k}_{\mathrm{eq},p}=\frac{{k}_{\mathrm{on},p}N[MT]}{{k}_{\mathrm{off},p}}=\frac{N[\,MT\,]}{{K}_{d,p}},\\ \frac{{\rho }_{h}}{(x-{\rho }_{p}-{\rho }_{h})}={k}_{\mathrm{eq},h}{{\rm{\Phi }}}_{h}({\rho }_{p},{\rho }_{h});\,{k}_{\mathrm{eq},h}=\frac{{k}_{\mathrm{on},h}N[MT]}{{k}_{\mathrm{off},h}}=\frac{N[MT]}{{K}_{d,h}}\,,\end{array}$$

where *K*_d,p_ and *K*_d,h_ are the dissociation constants related to the longitudinal “*p*” and the lateral “*h*” binding modes, respectively. The Φ_*p*_(*ρ*_*p*_, *ρ*_*h*_) and Φ_*h*_(*ρ*_*p*_, *ρ*_*h*_) represent the probabilities of inserting an additional Tau in “*p*” and “*h*” mode, respectively, on the MT lattice already covered at the equilibrium with a distribution of Taus at *ρ*_*p*_ and *ρ*_*h*_. Specifically, for the system under consideration (binding rules described above and illustration in Fig. 3) where a protofilament and a helix is treated as a homogeneous one-dimensional lattice of identical and independent point (of zero size) binding sites, the probabilities Φ_p_ and Φ_h_ for noncooperative binding of Tau-molecules are given by (see Sec. 5.2 for the derivation):

3 $$\{\begin{array}{c}{{\rm{\Phi }}}_{{\rm{p}}}({\rho }_{p},{\rho }_{h})=\frac{{[1-(1+{\sigma }_{p}){\rho }_{p}-(1+{\sigma }_{h}){\rho }_{h}]}^{1+{\sigma }_{p}}}{{(1-{\sigma }_{p}{\rho }_{p})}^{{\sigma }_{p}}},\\ {{\rm{\Phi }}}_{{\rm{h}}}({\rho }_{p},{\rho }_{h})=\frac{{[1-(1+{\sigma }_{h}){\rho }_{h}-(1+{\sigma }_{p}){\rho }_{p}]}^{1+{\sigma }_{h}}}{{(1-{\sigma }_{h}{\rho }_{h})}^{{\sigma }_{h}}}.\end{array}$$

By construction, Φ_i_(*ρ*_*p*_, *ρ*_*h*_) (*i* = *p*, *h*) satisfy the criteria: Φ_i_(*ρ*_*p*_ = 0, *ρ*_*h*_ = 0) = 1 for an empty MT lattice and Φ_i_(*ρ*_*p*,*s*_, *ρ*_*h*,*s*_) = 0 at the saturation coverages, 1 − (1 + *σ*_*p*_)*ρ*_*p*,*s*_ − (1 + *σ*_*h*_)*ρ*_*h*,*s*_ = 0, when the MT lattice is saturated with Tau-molecules.

At the end, the decoration of microtubules with Taus is described at the equilibrium by the system of coupled non-linear equations in Eq. (2) with insertion probabilities of Taus given in Eq. (3).

### Model outcomes

To explore and illustrate the richness of MT’s decorating model with Taus as we have just described above, we use the following characterizing observables (at the equilibrium):

- First and foremost is, *ρ* = *ρ*_*p*_ + *ρ*_*h*_, the total MT coverage with Tau-proteins (= [concentration of bound Taus]/[concentration of binding sites]). This experimentally accessible quantity can be measured using, for instance, equilibrium co-sedimentation experiments. As far we know, most of experiments measures *ρ* but not partial coverages *ρ*_*p*_ and *ρ*_*h*_. Solving Eqs. (2) with (3) provides access to the underlying structure of *ρ* that depends on 5 key parameters (see Table 1): the Tau binding sizes *σ*_*p*_ and *σ*_*h*_, the dissociation constants *K*_d,p_ and *K*_d,h_, and the Tau:tubulin-dimer ratio *x*.

**Table 1:** The kinetics of microtubule decoration with Taus is controlled by 7 key parameters.

	Symbol	Definition
Parameters	*σ*_*i*_	Size of Tau bound in the mode “*i* = *p*, *h*”
	*k*_on,i_	Tau on-rate (1/[concentration]/time) in the mode “*i* = *p*, *h*”
	*k*_off,i_	Tau off-rate (1/time) in the mode “*i* = *p*, *h*”
	*x*	Tau: tubulin-dimer ratio
Observables	*ρ*_*i*_	Microtubule coverage with Taus in the mode “*i* = *p*, *h*”
	*P*_*k*_(*r*)	Distribution of nearest neighbors along the direction *k* = ||, ⊥
	〈*r*_*k*_(*r*)〉	Mean nearest neighbors distance in the direction *k* = ||, ⊥
	*S*	Order parameter

At the equilibrium, the decoration involves only 5 key parameters: *σ*_*p*_, *σ*_*h*_, *x* and the two dissociation constants *K*_d,p_ = *k*_off,p_/*k*_on,p_ and *K*_d,h_ = *k*_off,h_/*k*_on,h_. The associated stoichiometry of Tau is, *ν*_*i*_ = 1/(1 + *σ*_*i*_). The main observables are the total microtubule coverage *ρ* = *ρ*_*p*_ + *ρ*_*p*_ and the distributions of nearest neighbors *P*_*k*_(*r*). Additional observables are the mean distance 〈*r*_*k*_(*r*)〉 separating two nearest neighbor bound Taus and the order parameter *S*.
- Second is the probability distribution of the nearest neighbor that provides the Tau-related spatial structure on the MT surface in the “*p*” and “*h*” modes, i.e., the structure of the decoration. To characterize the two-dimensional spatial structure associated to *ρ*, we consider two probability distributions *P*_∥_(*r*) and *P*_⊥_(*r*) of the nearest neighbor bound Taus along the protofilament and helix directions, respectively, where *r* is the unitless (in binding site unit = 8 *nm*) center-to-center distance separating two nearest-neighbors bound Tau’s (see Sec. 5.2.2 for details). Once *ρ*_*p*_ and *ρ*_*h*_ are determined (and *σ*_*p*_ and *σ*_*h*_ known), the distributions *P*_∥_(*r*) and *P*_⊥_(*r*) are calculated using Eq. (12) with Eq. (17). However, from an experimental point of view, it may turn out quite challenging to resolve the distribution of Tau-proteins along the MT helices, i.e., the lateral distribution *P*_⊥_(*r*). Therefore, we will only discuss the properties of *P*_∥_(*r*) that can be investigated from experimental data^39^ and leave *P*_⊥_(*r*) in the section Methods 5.2.
- Third is the order parameter, *S*, that characterizes the overall picture of spatial arrangements of Tau’s on the MT lattice. Likewise, once *ρ*_*p*_ and *ρ*_*h*_ are determined (and *σ*_*p*_ and *σ*_*h*_ known), the order parameter is calculated as, *S* = (1 + *σ*_*p*_)*ρ*_*p*_ − (1 + *σ*_*h*_)*ρ*_*h*_. By definition, −1 ≤ *S* ≤ +1, with *S* = +1 describes the case where all bound Tau’s are aligned along the protofilaments whereas *S* = −1 the one where the bound Tau’s are all aligned along the MT helices and *S* = 0 corresponds to the 50–50 situation.

To guide and circumscribe the exploration of the model in the space of 5 parameters (see Table 1) we consider the coverage curves in the phase space (*ρ*_*p*_, *ρ*_*h*_) in which each point of the curve corresponds to the total MT coverage as, *ρ* = *ρ*_*p*_ + *ρ*_*h*_. The physical space for the possible values of *ρ*_*p*_ and *ρ*_*h*_, [(0, 0) ≤ (*ρ*_*p*_, *ρ*_*h*_) ≤ (*ρ*_*p*,*s*_, *ρ*_*h*,*s*_)], is a rectangle triangle delimited by the horizontal *ρ*_*h*_ = 0 and vertical *ρ*_*p*_ = 0 axes and the saturation line, 1 − (1 + *σ*_*p*_)*ρ*_*p*_ − (1 + *σ*_*h*_)*ρ*_*h*_ = 0, originating from positivity condition of insertion probabilities, Φ_p_ ≥ 0 and Φ_h_ ≥ 0. The portraits of *ρ*_*h*_ as a function of *ρ*_*p*_ (obtained from the ratio of equations in Eq. (2)) is given by,

4 $$\frac{{\rho }_{p}}{{\rho }_{h}}=\kappa \frac{{(1-{\sigma }_{h}{\rho }_{h})}^{{\sigma }_{h}}}{{(1-{\sigma }_{p}{\rho }_{p})}^{{\sigma }_{p}}}{[1-(1+{\sigma }_{p}){\rho }_{p}-(1+{\sigma }_{h}){\rho }_{h}]}^{{\sigma }_{p}-{\sigma }_{h}},\,\kappa =\frac{{k}_{\mathrm{eq},{\rm{p}}}}{{k}_{\mathrm{eq},{\rm{h}}}}=\frac{{K}_{{\rm{d}},{\rm{h}}}}{{K}_{{\rm{d}},{\rm{p}}}}.$$

**Figure 4:**
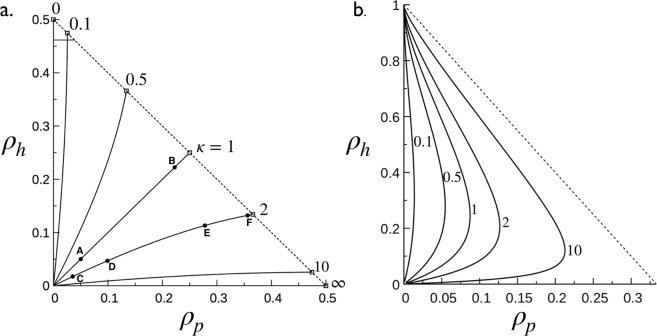
Coverage phase space of the microtubule decoration with Taus: *ρ*_*h*_ as a function of *ρ*_*p*_ for various values of *κ* (quoted numbers). Solid lines are obtained from Eq. (4) (panels a,b) and dashed lines represent the saturation line given by, 1 − (1 + *σ*_*p*_)*ρ*_*p*_ − (1 + *σ*_*h*_)*ρ*_*h*_ = 0, (in panel b, the saturation line reduces to the point (*ρ*_*p*_ = 0, *ρ*_*h*_ = 1)). Intersections between solid lines and the dashed line give the coordinates (*ρ*_*p*,*s*_, *ρ*_*h*,*s*_) at saturation. (**a**) Case of *σ*_*p*_ = *σ*_*h*_ = 1 (see Figs 2 and 3 for illustration). Points *A* and *B* on the line *κ* = 1 correspond to *x* = 0.15 and *x* = 10, respectively, with *k*_eq_ = 3. On the line *κ* = 2, points *C* and *D* correspond to *x* = 0.15 with *k*_eq_ = 0.66 and *k*_eq_ = 66, respectively, and *E* and *F* to *x* = 10 with *k*_eq_ = 0.66 and *k*_eq_ = 66, respectively. (**b**) Case of *σ*_*p*_ = 2 and *σ*_*h*_ = 0. Adapted from the manuscript of J.H.’s thesis^58^.

As shown in Fig. 4, *κ* is the key organizing parameter of portraits *ρ*_*h*_ *vs ρ*_*p*_ parameterized by the *σ*’s. When *σ*_*p*_ = *σ*_*h*_, Eq. (4) shows that *ρ*_*h*_ = *ρ*_*p*_ is the trivial solution for *κ* = 1 and that the portraits for *κ* ≠ 1 are symmetric about the *κ* = 1 trajectory, i.e., *ρ*_*h*_(*ρ*_*p*_, *κ*) = *ρ*_*p*_(*ρ*_*h*_, 1/*κ*), as illustrated in Fig. 4a for *σ*_*p*_ = *σ*_*h*_ = 1. In contrast, when *σ*_*p*_ ≠ *σ*_*h*_, the general trend of portraits is quite different from that of *σ*_*p*_ = *σ*_*h*_ in the sense that *ρ*_*h*_ as a function of *ρ*_*p*_ is now bi-valued with an extremum and all portrait lines converge to the saturation coordinates (*ρ*_*p*,*s*_ = 0, *ρ*_*h*,*s*_ = 1/(1 + *σ*_*h*_)) for *σ*_*p*_ > *σ*_*h*_ or (*ρ*_*p*,*s*_ = 1/(1 + *σ*_*p*_), *ρ*_*h*,*s*_ = 0) for *σ*_*p*_ < *σ*_*h*_, i.e., the system converges to the highest stoichiometry at the saturation as illustrated in Fig. 4b for *σ*_*p*_ = 2 and *σ*_*h*_ = 0.

We now consider in detail two cases according to *κ* to gain more insights into the MT decoration.

#### Single binding mode

**Figure 5:**
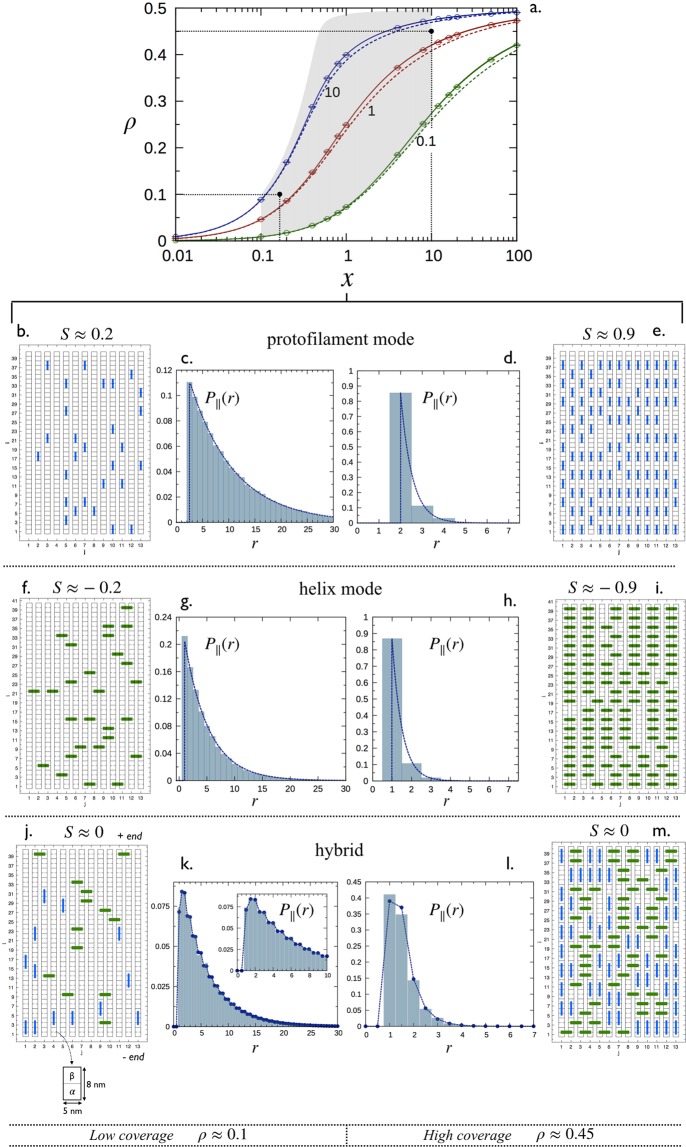
Decoration of a microtubule with Tau proteins. (**a**) Saturation curves: total coverage *ρ* at equilibrium state as a function of *x* for *σ*_*p*_ = *σ*_*h*_ = 1. Point data (circles) represent results from Monte Carlo simulations in the limit case *κ* → +∞ (mode “*p*”) with *k*_eq,p_ = 0.1, 1 and 10. Solid and dashed lines correspond to numerical solutions of Eq. (2) in the limit cases *κ* → +∞ (for *ρ*) and *κ* = 1 (for *ρ*_*p*_ and *ρ*_*p*_ with *ρ* = *ρ*_*p*_ + *ρ*_*p*_), respectively (see Sec. 5.4). The grayed zone defines the domain of coverage consistent with the axonal conditions. The zone has been obtained using the two ranges 0.1 ≤ *x* ≤ 10 and 0.1 ≤ *k*_eff_ ≤ 10^3^ (see Sec. 5.1 for details). The two black dots correspond to *A* and *B* in Fig. 4a. (**b**–**m**) Snapshots and their corresponding distributions *P*_||_(*r*) for a single binding mode in (**b**–**i**) and two binding modes with *κ* = 1 in (**j**–**m**). In each case, the decoration is characterized for low *ρ* ≈ 0.1 (*x* = 0.15), and high coverages, *ρ* ≈ 0.45 (*x* = 10). Histograms for the distributions of nearest neighbors in (**c**,**d**,**g**,**h**,**k**,**l**) have been calculated from Monte Carlo simulations (see Sec. 5.3). Dashed lines in (**c**,**d**,**g**,**h**,**k**,**l**) correspond to theoretical distributions obtained using Eq. (6) for the mode “*p*” in (**c**,**d**) and the mode “*h*” in (**g**,**h**), and Eq. (12) with Eq. (17) for the two binding modes in (**k**,**l**). Adapted from the manuscript of J.H.’s thesis^58^.

The single binding mode corresponds to the case when Tau-molecules can bind only either in the mode “*p*” (*κ* → +∞: protofilament binding mode) or in the mode “*h*” (*κ* → 0: helix binding mode). In each case the MT decoration is controlled by 3 key parameters (see Table 1): the Tau binding size *σ*_*i*_ (or stoichiometry *ν*_*i*_ = 1/(1 + *σ*_*i*_)), the dissociation constant *K*_d,i_ and the Tau:tubulin-dimer ratio *x*. In this limit, *ρ*_*j*_ = 0, corresponding to the either x-axis (for “*p*”) or y-axis (for “*h*”) in the phase space in Fig. 4a, and the total coverage, *ρ* = *ρ*_*i*_ (*i* ≠ *j*), lies between 0 and the saturation *ρ*_*s*_ = 1/(1 + *σ*_*i*_) ≡ *ν*_*ii*_, depending of *k*_eq,i_ and *x*. Figure 5 synthetically illustrates the general picture corresponding to this case. The MT decoration is characterized as follows:

- *MT coverage*: Fig. 5a shows numerical solutions (solid lines) of Eq. (2) along with simulations results (data points) of *ρ* as a function of *x* for *σ*_*i*_ = 1 and various *k*_eq,i_. At low *x*, the coverage *ρ* linearly increases with *x* then deviates from linearity and slowly reaches the saturation *ρ*_*s*_ ≡ *ν*_*ii*_ = 0.5 at high *x*.

The diluted regime, corresponding to a low MT coverage *ρ* ≪ 1, is especially relevant to the context of axons^29^. In this case, the coverage is given by (see Supplementary Information, Sec. S1):

5 $$\rho =\frac{1}{2}[(\frac{1+{{k}_{\mathrm{eq},{\rm{i}}}}^{-1}}{1+2{\sigma }_{i}}+x)-\sqrt{{(\frac{1+{{k}_{\mathrm{eq},{\rm{i}}}}^{-1}}{1+2{\sigma }_{i}}+x)}^{2}-\frac{4x}{1+2{\sigma }_{i}}}],\,i=p,h.$$

As a consequence, the two limits *κ* → +∞ (protofilament binding mode) and *κ* → 0 (helix binding mode) cannot be distinguished in terms of microtubule coverage.

• *Distribution of Tau spacing:* The nearest neighbor distribution, *P*_∥,*i*_(*r*), along the protofilaments and the associated first moment, 〈*r*_∥,*i*_〉, for *i* = *p*, *h* modes are given by (Sec. 5.2.2):

6 $${P}_{\parallel ,i}(r)=\{\begin{array}{ll}\frac{\rho }{1-{\sigma }_{p}\rho }{(\frac{1-(1+{\sigma }_{p})\rho }{1-{\sigma }_{p}\rho })}^{r-(1+{\sigma }_{p})} & r-(1+{\sigma }_{p})=0,1,2,\cdots ,\,i=p,\\ (1+{\sigma }_{h})\rho {[1-(1+{\sigma }_{h})\rho ]}^{r-1} & r-1=0,1,2,\cdots ,\,i=h,\\ 0 & \mathrm{otherwise}\,.\end{array}$$

and,

7 $$\langle {r}_{\parallel ,i}\rangle =\{\begin{array}{cc}\frac{1}{\rho } & i=p,\\ \frac{1}{(1+{\sigma }_{h})\rho } & i=h.\end{array}$$

Note that 〈*r*_∥,*p*_〉 is independent of *σ*_*p*_ while 〈*r*_∥,*h*_〉 decreases with *σ*_*h*_. For example, for a minimal coverage, *ρ* = 2/*h*, of two Taus per protofilament of length *h*, and a typical microtubule of length 5 *μm*, corresponding to *h* = 5 *μm*/8 *nm* = 625, we found that the mean Tau spacing, 〈*r*_∥,*p*_〉 = 8 *nm*/*ρ* = 2.5 *μm* (in real units) when bound in mode “*p*” and 〈*r*_∥,*h*_〉 = 8 *nm*/[(1 + *σ*_*h*_)*ρ*] = 1.25 *μm* when bound in mode “*h*” with *σ*_*h*_ = 1.

• *Spatial arrangement of Taus:* To illustrate the spatial arrangement of Taus on the MT surface, we consider two contrasted configurations at low (*x* = 0.15, *ρ* ≈ 0.1) and high (*x* = 10, *ρ* ≈ 0.45) coverages at the same equilibrium constant *k*_eq,i_ = 3. The two configurations are indicated by filled circles in Fig. 5a and snapshots of Tau arrangement on MT surface with associated Tau spacing distribution *P*_∥,*i*_(*r*) are displayed in Fig. 5b–e (for *i* = *p*, protofilament binding mode) and Fig. 5f–i (for *i* = *h*, helix binding mode); snapshots and histogram in *P*_∥,*i*_(*r*) are from simulations and lines are from Eq. (6)).

At low coverages, corresponding to an order parameter *S* ≈ 0.2 for *i* = *p* and *S* ≈ −0.2 for *i* = *h*, there is no apparent spatial organization of Taus (Fig. 5b,f) and their spacing distributions are an exponential decay (Fig. 5c,g) with a maximum probability ≈10% at 8 *nm* × (1 + *σ*_*p*_) and 8 *nm*, respectively. In contrast, at higher coverages with *S* ≈ ±0.9, there is a clear spatial order in the protofilament (Fig. 5e) and helix (Fig. 5i) directions and the nearest neighbor spacing distributions in Fig. 5d and h show a sharp exponential decay with maximum probabilities ≈85%.

At the saturation limit, *ρ* = 1/2 and *S* = ±1; all the Tau-proteins are perfectly aligned along the protofilaments or the helices with a nearest neighbor distribution given by a Dirac delta function centered on modal positions. By analogy with crystalline liquids, the configurations of Fig. 5b,f can be regarded as a nematic-type phase, whereas those of Fig. 5e,i as a smectic-type phase.

#### Protofilament and helix binding modes

When both two Tau binding modes can occur, the MT decoration involves 5 key parameters (see Table 1): two Tau binding sizes *σ*_*p*_ and *σ*_*h*_, two dissociation constants *K*_d,p_ and *K*_d,h_, and the Tau:tubulin-dimer ratio *x*. It thus follows that 4 contrasted situations can be distinguished: *κ* = 1 with *σ*_*p*_ = *σ*_*h*_ and *σ*_*p*_ ≠ *σ*_*h*_, and *κ* ≠ 1 with *σ*_*p*_ = *σ*_*h*_ and *σ*_*p*_ ≠ *σ*_*h*_.

**Figure 6:**
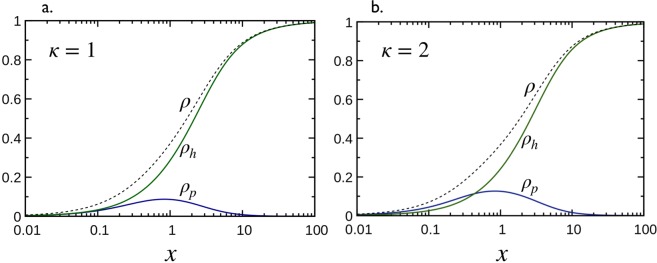
Decoration of microtubules with Tau proteins for *σ*_*p*_ = 2 and *σ*_*h*_ = 0. Coverages *ρ*_*p*_, *ρ*_*h*_ (solid lines) and the total coverage *ρ* = *ρ*_*p*_ + *ρ*_*h*_ (dashed lines) as a function of *x* for *k*_eq,p_ = *k*_eq,h_ = 1 (i.e., *κ* = 1) in (**a**) and *k*_eq,p_ = 2, *k*_eq,h_ = 1 (i.e., *κ* = 2) in (**b**). Solid lines are obtained from the numerical solutions of Eq. (2) with Φ’s given in Eq. (3).
- *Distribution of Tau spacing:* Inspection of Fig. 5j–m (snapshots and histogram in *P*_∥_(*r*) are from simulations and lines are from Eqs (12) and (17)) shows that allowing two binding modes for Taus effectively impact the spacing distributions. As can be seen in Fig. 5k,l, *P*_∥_(*r*) in Eq. (12) is no longer a single exponential distribution but rather a summation of exponential decays with *P*_∥,*ij*_(*r*) given in Eq. (17). The difference in the *P*_∥_(*r*) shape, in comparison with that of the single binding modes, is particularly noticeable for configurations closed to the saturation, as illustrated in Fig. 5l.
- *Spatial arrangement of Taus:* The low (*x* = 0.15, *ρ* ≈ 0.1) and high (*x* = 10, *ρ* ≈ 0.45) coverage configurations considered above in single binding modes correspond here to the points *A* and *B* (with *σ*_*p*_ = *σ*_*h*_ = 1 and *k*_eff_ = 3) along the linear trajectory *ρ*_*h*_ = *ρ*_*p*_ in the phase space in Fig. 4a. As *σ*_*p*_ = *σ*_*h*_, both configurations *A* and *B* are characterized by an order parameter *S* ≈ 0, i.e., there is on average the same amount of Taus bound in “*p*” and “*h*” modes as can be seen in the snapshots of Fig. 5j,m. However, as shown in Fig. 4b for *κ* = 1 and *σ*_*p*_ = 2 and *σ*_*h*_ = 0 the curve *ρ*_*h*_ *vs ρ*_*p*_ can be found below or above the line *ρ*_*h*_ = 3*ρ*_*p*_ depending on *k*_eff_ and *x*. Therefore, the order parameter can be found *S* < 0, *S* = 0 and *S* > 0 depending on *k*_eff_ and *x*. At the saturation, *S* < 0 for *σ*_*p*_ > *σ*_*h*_ and vice versa.

• **Identical dissociation constants:** ***κ*** = 1

- *MT coverage:* The phase spaces in Fig. 4 show two different trajectories of *κ* = 1 (panels *a* and *b*). As can be seen, and already emphasized in the text below Eq. (4), the portraits are linear, *ρ*_*h*_ = *ρ*_*p*_ for *σ*_*p*_ = *σ*_*h*_, and non-linear otherwise, and coordinates (*ρ*_*p*_, *ρ*_*h*_) along the trajectories depends both on *K*_d,p_ = *K*_d,h_ and *x*. Numerical results for the total coverage *ρ* as a function of *x*, for *σ*_*p*_ = *σ*_*h*_ = 1 and three values of *k*_eff_ = *k*_eq,p_ + *k*_eq,h_ = 0.1, 1 and 10, are shown by dashed lines in Fig. 5a. Clearly, the behaviors of *ρ* as a function of *x* are all very similar both in single (*κ* → +∞ and *κ* → 0 limits) and two binding modes. However, the situation is quite different when *σ*_*p*_ ≠ *σ*_*h*_ (see Fig. 6). Indeed, Fig. 4b for *κ* = 1 and *σ*_*p*_ = 2 and *σ*_*h*_ = 0 shows that *ρ*_*h*_ monotonically increases from zero to saturation around *ρ*_*h*_ = 1 while *ρ*_*p*_ (<*ρ*_*h*_) increases from zero reaches a maximum and decreases when approaching saturation conditions. This indicates that below the saturation conditions, the system is bi-phasic and admits two equilibrium coverages with identical *ρ*_*p*_ and two different *ρ*_*h*_. At saturation, the system becomes mono-phasic involving only the binding mode with smaller *σ* (higher stoichiometry).

• **Non-identical equilibrium constants:** ***κ*** ≠ 1

To illustrate the MT decoration in the situation of non-identical equilibrium constants of binding modes, we consider the case *κ* = 2, shown in the phase space in Fig. 4a for *σ*_*p*_ = *σ*_*h*_ = 1. The 4 points along the trajectory correspond to the following configurations: *C* = (*x* = 0.15, *ρ* ≈ 0.05, *k*_eff_ = 0.66) and *D* = (*x* = 0.15, *ρ* ≈ 0.15, *k*_eff_ = 66) for low coverage, and *E* = (*x* = 10, *ρ* ≈ 0.39, *k*_eff_ = 0.66) and *F* = (*x* = 10, *ρ* ≈ 0.48, *k*_eff_ = 66) for high coverage.

**Figure 7:**
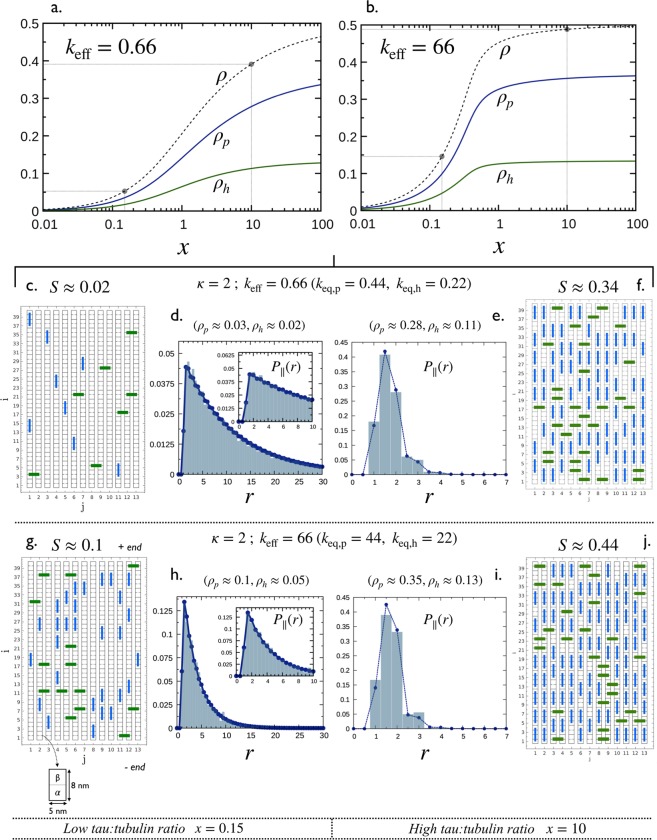
Decoration of a microtubule by Tau for the case *κ* = 2 (i.e., *k*_eq,p_ = 2*k*_eq,h_) and *σ*_*p*_ = *σ*_*h*_ = 1. (**a**,**b**) partial coverages *ρ*_*p*_, *ρ*_*h*_ and the total coverage *ρ* = *ρ*_*p*_ + *ρ*_*h*_ as a function of the ratio, *x*, for effective equilibrium constants *k*_eff_ = 0.66 and 66, respectively. Solid lines correspond to numerical solutions of Eq. (2). In both cases, results corresponding to *C* and *D* (*x* = 0.15) and *E* and *F* (*x* = 0.10) in Fig. 4 are highlighted in black dots. This leads to 4 configurations: *C*, *D*, *E* and *F* (see Fig. 4a) with typical snapshots and averaged longitudinal spacing distributions, *P*_||_(*r*) shown in (**c**–**j**). Histograms in (**d**,**e**,**h**,**i**) are calculated from Monte Carlo simulations (see Sec. 5.3) while dashed lines with points are obtained using Eq. (12) with Eq. (17). Adapted from the manuscript of J.H.’s thesis^58^.
- *Distribution of Tau spacing:* Snapshots of MT decoration and associated nearest neighbor distributions for the 4 configurations *C*, *D*, *E* and *F* (filled circle points in Fig. 7a,b) are shown in Fig. 7c–j (snapshots and histogram in *P*_||_ are from simulations and lines are from Eqs (12) and (17)).

- *MT coverage:* Fig. 7a,b show the partial and total coverage as a function of *x*, for *σ*_*p*_ = *σ*_*h*_ = 1 and *k*_eff_ = 0.66 and 66. Configurations *C* and *E* are represented by the two filled circle points on *ρ vs x* in Fig. 7a, and *D* and *F* by the filled circle points on the same curve in Fig. 7b. Coverages *ρ*_*p*_ and *ρ*_*h*_ linearly increase with *x* at low *x* and slowly reach their saturations ${\rho }_{p,s}=[\sqrt{3}-1]/2\approx 0.36$ and ${\rho }_{h,s}=[1-\sqrt{3}]/2\approx 0.13$ (as predicted in Eq. (S3) in the Supplementary Information, Sec. S1) at high. In any cases, we have *ρ*_*p*_ > *ρ*_*h*_.

The shape of the distributions in Fig. 7d,h, corresponding to the low coverage configurations *C* and *E*, are very similar to that in Fig. 5k for *κ* = 1 (i.e., configuration *A* in Fig. 4b) with a multi-exponential decay, while the shape for high density configurations *D* and *F* exhibit significant differences. Indeed, the main peak of *P*_∥_(*r*) in Fig. 7e,i is centered at *r* = 1.5, corresponding to closed packing between Taus bound in longitudinal “*p*” (in blue) and lateral “*h*” (in green) modes. In addition, the peak centered at *r* = 2, corresponding to closed packing between Taus bound in “*p*” mode, is higher than that centered at *r* = 1 for Taus bound in “*h*” mode. This is because for *κ* = 2 there are more Taus bound in “*p*” mode than those bound in “*h*” modes, i.e, *ρ*_*p*_ > *ρ*_*h*_.

- *Spatial arrangement of Taus:* As *ρ*_*p*_ > *ρ*_*h*_ and that the curve *κ* = 2 in Fig. 4b is below the line *ρ*_*h*_ = *ρ*_*p*_, all configurations in this case are characterized by an order parameter *S* > 0 as shown in Fig. 7c,f,g,j.

Conducting a similar analysis for *κ* = 2, *σ*_*p*_ = 2 and *σ*_*h*_ = 0, as shown in the phase space in Fig. 4b, leads to similar observations emphasized above for the case of *κ* = 1 (as illustrated in Fig. 6).

## Conclusion

Our main motivation in developing this work has been the paramount importance of the interactions between Tau proteins and microtubules in axons. In particular, Tau molecules play a crucial role in many neurodegenerative diseases referred to as *Tauopathies*. Our goal was to study and describe how a stabilized microtubule is decorated by a population of Tau in terms of coverage and spatial distributions of Taus on the microtubule outer surface. Based on published experimental evidences, we have developed a model of Tau-microtubule interaction in which Tau proteins can reversibly bind to the microtubule lattice either along a protofilament (mode “*p*”) on two *αβ*-tubulin dimers or laterally (mode “*h*”) on two adjacent dimers as shown in Fig. 3. We show that the decoration of microtubules with Taus is described at the equilibrium by the system of coupled non-linear equations in Eqs (2) and (3) whose solution provides the partial coverages, *ρ*_*p*_ and *ρ*_*h*_, such the total microtubule coverage with Tau’s is given by, *ρ* = *ρ*_*p*_ + *ρ*_*h*_,

Within this framework, the decoration of microtubules with Tau’s is controlled by 5 key parameters: the Tau binding stoichiometries (related to Tau’s sizes *σ*_*p*_ and *σ*_*h*_,) in modes “*p*” and “*h*”, the dissociation constants in modes “*p*” and “*h*” (*K*_d,p_ and *K*_d,h_) and the Tau:tubulin-dimer ratio *x*. The line portraits in the phase space, {*ρ*_*p*_, *ρ*_*h*_}, of the microtubule decoration (see Fig. 4) are defined by the ratio, *κ* = *K*_d,h_/*K*_d,p_, of dissociation constants, parameterized by sizes *σ*_*p*_ and *σ*_*h*_, and the location along the lines is controlled by *x* and the effective equilibrium constant, *k*_eff_ = *k*_eq,p_ + *k*_eq,h_. Each point in the phase diagram corresponds to a distribution of Taus attached on the microtubule wall which is characterized by the coverages *ρ*_*p*_ and *ρ*_*h*_ and the averaged distribution for the longitudinal spacing of Tau’s, *P*_∥_(*r*). A microtubule decorated with Taus bound in a single mode (“*p*” or “*h*”) exhibits a single exponential decay for *P*_∥_(*r*) (see Fig. 5c,d,g,h) while for the mixed case of Taus bound in two modes (“*p*” and “*h*”), *P*_∥_(*r*) exhibits a multi-exponential behavior (see Figs 5k,l and 7d,e,h,i).

For experimental purposes, the decoration model described above can be used as a theoretical framework for interpreting and analyzing, for example, binding data from co-sedimentation assays and distributions for the longitudinal spacing of Tau’s using quick-frozen, deep-etched suspension of microtubules as in^39^.

Although this work can already be used for realistic experimental situations, it can be further extended in several directions including the possible role associated to the Tau’s binding modes “*p*” and “*h*”, the shape or spatial extension of bound Tau (this proteins being highly dynamic even when bound to microtubules), the effect of the microtubule curvature on the decoration and the heterogeneous microtubule lattice with GTP and GDP tubulins (heterogeneous binding sites).

Regarding the binding modes “*p*” and “*h*”, since the Tau binding domain involves three or four repeats^45^ capable of binding independently to a *α* or a *β* monomer^28^, it can be assumed that Tau will probably adopt a elongated shape form when bound along a protofilament and a more crushed form when bound through protofilaments. Therefore, both Tau conformations longitudinally or laterally may be associated with distinct biological behaviors as suggested by ref.^38^. For instance, longitudinally bound Taus could act as bridges between microtubules to form the microtubule network, while those bound in lateral mode could prevent microtubules from catastrophe events and thus stabilize them. Likewise, the helical geometry of the microtubule, that is, its curvature, could be expected to affect the laterally bound Tau, thus modifying the *κ* ratio of dissociation constants.

Finally, it would be very useful to generalize the model and approach developed in this work to non-stabilized and dynamic microtubule lattice in order to study the effect of Tau on the dynamic instability of microtubules.

## Methods

### Binding parameter estimates: ***k***_**eff**_ and ***x***

In human axons, the total concentration of Tau, [Tau], was found between ~1% and 20% of the total tubulin-dimer concentration (both free and polymerized)^46^. In addition, more than 80% of the tubulins in the squid giant axon was found in the free form (i.e., not polymerized)^47^. In this specific case, the total concentration of tubulin is 5 times greater than the polymerized one i.e., [Tub_tot_] = 5[Tub_poly_]. Throughout this work, we chose to work with conditions, 5 ≤ [Tub_tot_]/[Tub_poly_] ≤ 50 corresponding to a Tau:tubulin-dimer ratio, 0.1 ≤ *x* = [Tau]/[Tub_poly_] ≤ 10. The effective equilibrium constant can be estimated using the relation *k*_eff_ = [Tub_poly_]/*K*_d_ ≡ [Tau]/(*x* × *K*_*d*_) where *K*_*d*_ is the dissociation constant. Reported *K*_*d*_ values vary by more than two orders of magnitude from ~0.01  *μ*M to ~1 *μ*M^28,29,35,42,43,48–52^. Therefore, with a typical concentration of ~1–2 *μ*M for Tau in axons^53,54^ and with the estimated ranges for *x* and *K*_*d*_, we end up with the range, 0.1 ≤ *k*_eff_ ≤ 10^3^.

### Mathematical derivations

#### Insertion probabilities

The insertion probabilities Φ_p_ and Φ_h_ are obtained from, ${{\rm{\Phi }}}_{k}={n}_{\mathrm{add},k}/{\ell }_{k}$, where ${\ell }_{k}$ is the total number of lattice sites along the *k* direction (${\ell }_{k}=h$ and ${\ell }_{k}=p$ for *k* = ∥ (protofilament axis) and *k* = ⊥ (helix axis), respectively) and *n*_add,__*k*_ is the mean number of distinct ways for adding a Tau-molecule of size *σ*_*k*_ along the *k* direction (*σ*_*k*_ = *σ*_*p*_ and *σ*_*k*_ = *σ*_*h*_ for *k* = ∥ and *k* = ⊥, respectively). For a given number *n*_*k*_ of Taus bound along the *k* direction corresponds a total of *n*_gap,__*k*_ = *n*_*k*_ + 1 gaps, each of size *g* with a probability given by the gap distribution *f*_*k*_(*g*) (see the Supplementary Information, Sec. S2.A). Therefore, *n*_add,__*k*_ can be obtained as,

8 $${n}_{\mathrm{add},k}={n}_{\mathrm{gap},k}\times {\delta }_{k}=({n}_{k}+1)\times {\delta }_{k},$$

where *δ*_*k*_ counts the fraction of configurations allowing to accommodate a particle of size *σ*_*k*_ within each gaps. Following the approach in^55^, *δ*_*k*_ is given by,

9 $${\delta }_{k}=\mathop{\sum }\limits_{g=1+{\sigma }_{k}}^{{g}_{{\rm{m}},k}}\,(g-{\sigma }_{k}){f}_{k}(g)={f}_{k}(1+{\sigma }_{k})+2{f}_{k}(2+{\sigma }_{k})+\mathrm{...}+({g}_{{\rm{m}},k}-{\sigma }_{k}){f}_{k}({g}_{{\rm{m}},k}),$$

Eq. (9) indicates that for a particle of size *σ*_*k*_, there is one way of inserting that particle into a gap of size 1 + *σ*_*k*_ (with probability, *f*_*k*_(1 + *σ*_*k*_)), two ways into a gap of size 2 + *σ*_*k*_ (with probability, *f*_*k*_(2 + *σ*_*k*_)), and so on up to the maximum physical gap size *g*_m,__*k*_. The insertion probability is therefore given by,

10 $${{\rm{\Phi }}}_{k}=({\rho }_{k}+\frac{1}{{\ell }_{k}})\times [{f}_{k}(1+{\sigma }_{k})+2{f}_{k}(2+{\sigma }_{k})+\mathrm{...}+({g}_{{\rm{m}},k}-{\sigma }_{k}){f}_{k}({g}_{{\rm{m}},k})].$$

In the limit of a very long lattice (i.e., ${\ell }_{k}\to \infty$), Φ_*k*_ in Eq. (10) reduces to,

11 $${{\rm{\Phi }}}_{k}={\rho }_{k}\mathop{\sum }\limits_{g=1+{\sigma }_{k}}^{+\infty }\,(g-{\sigma }_{k}){f}_{k}(g)={\rho }_{k}(1-{u}_{k})\mathop{\sum }\limits_{g=1+{\sigma }_{k}}^{+\infty }\,(g-{\sigma }_{k})\,{{u}_{k}}^{g}={\rho }_{k}(\frac{{u}_{k}^{1+{\sigma }_{k}}}{1-{u}_{k}}).$$

**Figure 8:**
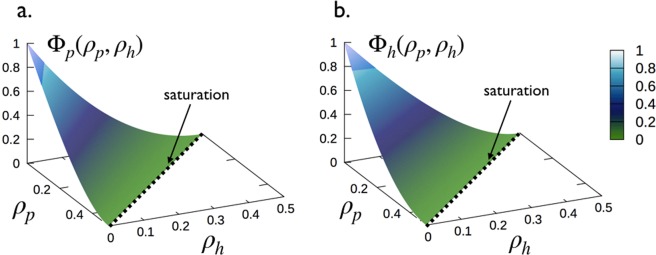
Surface plots of Φ_p_ and Φ_h_ in Eq. (3) as a function of *ρ*_*p*_ and *ρ*_*h*_ for *σ*_*p*_ = *σ*_*h*_ = 1. Dashed lines represent the saturation given by, 1 − (1 + *σ*_*p*_)*ρ*_*p*_ − (1 + *σ*_*h*_)*ρ*_*h*_ = 0. Adapted from the manuscript of J. H.’s thesis^58^.

The Φ_p_ and Φ_h_ in Eq. (3) have been obtained by using *f*_*k*_(*g*) (and thus, *ρ*_*k*_ and *u*_*k*_) derived in the Supplementary Information, Sec. S2.A. A graphical representation of Φ_p_ and Φ_h_ for *σ*_*p*_ = *σ*_*h*_ = 1 are shown in Fig. 8.

#### Distribution of nearest neighbors and associated first moment

We denote by *P*_∥_(*r*) and *P*_⊥_(*r*) the probability distributions of nearest neighbors bound Tau-proteins along the protofilament direction (∥) and along the helix direction (⊥), respectively, (see Fig. 1) where *r* is the unitless (in binding site unit = 8 *nm*) center-to-center distance separating two nearest-neighbors bound Tau’s. The probabilities *P*_*k*_(*r*) are given as,

12 $${P}_{k}(r)=\mathop{\sum }\limits_{i=1}^{2}\,\mathop{\sum }\limits_{j=1}^{2}\,{z}_{k,i}{z}_{k,j}\,{P}_{k,ij}(r),\,{\rm{with}}\,\{1\equiv p;\,2\equiv h\}\,{\rm{and}}\,k=\{\parallel ,\perp \},$$

where *P*_*k*,*ij*_(*r*) are the partial distributions of nearest neighbors (i.e., between two Tau’s bound in “*i* = *h*, *p*” and “*j* = *h*, *p*” modes) such that, $\mathop{\sum }\limits_{r=0}^{\infty }\,{P}_{k,ij}(r)=1$, and *z*_*k*,*i*_, the fractions of Tau’s bound in “*i*” mode counted in the direction *k*, are given by,

13 $${z}_{k,p}=\frac{{\rho }_{k,p}}{{\rho }_{k,p}+{\rho }_{k,h}}\,{\rm{and}}\,{z}_{k,h}=1-{z}_{k,p}.$$

The *ρ*_*k*,*i*_ are the directional coverage (at equilibrium) of Tau bound in “*i*” mode counted in the direction *k* (see the Supplementary information, Sec. S2.A):

14 $$(\begin{array}{l}{\rho }_{\parallel ,p}={\rho }_{p}\,\mathrm{and}\,{\rho }_{\parallel ,h}=(1+{\sigma }_{h}){\rho }_{h}\,,\\ {\rho }_{\perp ,h}={\rho }_{h}\,\mathrm{and}\,{\rho }_{\perp ,p}=(1+{\sigma }_{p}){\rho }_{p}\,.\end{array}$$

The *P*_*k*,*ij*_(*r*) are derived from the distribution of gaps as follows,

15 $${P}_{k,ij}(r)=\sum _{g}\,{\delta }_{g-(r-{r}_{k,ij})}\,{f}_{k}(g){=}_{\mathrm{lim}h,p\gg 1}(\begin{array}{ll}(1-{u}_{k})\,{{u}_{k}}^{r-{r}_{k,ij}} & \mathrm{for}\,r-{r}_{k,ij}=0,1,2,\cdots ,\\ \,0 & \mathrm{otherwise}\,.\end{array}\,,$$

where the matrix elements *r*_*k*,*ij*_, correspond to the close packing center-to-center distance between Tau’s bound in “*i*” and “*j*” modes along the direction *k*, are given by

16 $${r}_{k}=(\begin{array}{ll}{r}_{k,pp} & {r}_{k,ph}\\ {r}_{k,hp} & {r}_{k,hh}\end{array})=(\begin{array}{ll}(1+{\sigma }_{p}){\delta }_{k,\parallel }+{\delta }_{k,\perp } & 1+\frac{{\sigma }_{k}}{2}\\ 1+\frac{{\sigma }_{k}}{2} & (1+{\sigma }_{h}){\delta }_{k,\perp }+{\delta }_{k,\parallel }\end{array}).$$

Using *u*_*k*_ derived in the Supplementary Information, Eq. (S14) in Sec. S2.A and (15) leads to,

17 $${P}_{k,ij}(r){=}_{\mathrm{lim}h,p\gg 1}\{\begin{array}{ll}\frac{{\rho }_{p}+(1+{\sigma }_{h}){\rho }_{h}}{1-{\sigma }_{p}{\rho }_{p}}\,{(\frac{1-(1+{\sigma }_{p}){\rho }_{p}-(1+{\sigma }_{h}){\rho }_{h}}{1-{\sigma }_{p}{\rho }_{p}})}^{r-{r}_{\parallel ,ij}}, & r-{r}_{\parallel ,ij}=0,1,2,\cdots ,\,\,k=\parallel \\ \frac{{\rho }_{h}+(1+{\sigma }_{p}){\rho }_{p}}{1-{\sigma }_{h}{\rho }_{h}}{(\frac{1-(1+{\sigma }_{p}){\rho }_{p}-(1+{\sigma }_{h}){\rho }_{h}}{1-{\sigma }_{h}{\rho }_{h}})}^{r-{r}_{\perp ,ij}}, & r-{r}_{\perp ,ij}=0,1,2,\cdots ,\,k=\perp \\ 0 & \mathrm{otherwise}\,.\end{array}$$

The mean distance between two Tau-molecules along a *k* direction is obtained as,

18 $$\begin{array}{rcl}\langle {r}_{k}\rangle  & = & \mathop{\sum }\limits_{r=0}^{+\infty }\,r\,{P}_{k}(r)\\  & = & \mathop{\sum }\limits_{i,j=1}^{2}\,{z}_{k,i}{z}_{k,j}\mathop{\sum }\limits_{r=0}^{+\infty }\,r\,{P}_{k,ij}(r)\\  & = & \mathop{\sum }\limits_{i,j=1}^{2}\,{z}_{k,i}{z}_{k,j}\{\frac{{u}_{k}}{1-{u}_{k}}+{r}_{k,ij}\}\\  & = & \frac{{u}_{k}}{1-{u}_{k}}+\mathop{\sum }\limits_{i=1}^{2}\,\mathop{\sum }\limits_{j=1}^{2}\,{z}_{k,i}{z}_{k,j}\,{r}_{k,ij}\end{array}$$

which, in the limit *h* ≫ 1 and *p* ≫ 1, reduces to:

19 $$\langle {r}_{k}\rangle =\{\begin{array}{cc}\frac{1-(1+{\sigma }_{p}){\rho }_{p}-(1+{\sigma }_{h}){\rho }_{h}}{{\rho }_{p}+(1+{\sigma }_{h}){\rho }_{h}}+\frac{(1+{\sigma }_{p}){\rho }_{p}^{2}+(2+{\sigma }_{p})(1+{\sigma }_{h}){\rho }_{p}{\rho }_{h}+{(1+{\sigma }_{h})}^{2}{\rho }_{h}^{2}}{{[{\rho }_{p}+(1+{\sigma }_{h}){\rho }_{h}]}^{2}}, & k=\parallel ,\\ \frac{1-(1+{\sigma }_{p}){\rho }_{p}-(1+{\sigma }_{h}){\rho }_{h}}{(1+{\sigma }_{p}){\rho }_{p}+{\rho }_{h}}+\frac{{(1+{\sigma }_{p})}^{2}{\rho }_{p}^{2}+(2+{\sigma }_{h})(1+{\sigma }_{p}){\rho }_{p}{\rho }_{h}+(1+{\sigma }_{h}){\rho }_{h}^{2}}{{[(1+{\sigma }_{p}){\rho }_{p}+{\rho }_{h}]}^{2}}, & k=\perp .\end{array}$$

Note that, ${P}_{k,ij}(r)={\delta }_{r,{r}_{k,ij}}$ and 〈*r*_∥_〉 = 1/(1 − *σ*_*p*_*ρ*_*p*,*s*_) and 〈*r*_⊥_〉 = 1/(1 − *σ*_*h*_*ρ*_*h*,*s*_), at the saturation limit, 1 − (1 + *σ*_*p*_)*ρ*_*p*,*s*_ − (1 + *σ*_*h*_)*ρ*_*h*,*s*_ = 0.

### Monte carlo simulations

Stochastic simulations of the binding process described in 3.1 were performed for a two dimensional lattice of 615 × 13 ≈ 8000 sites corresponding to a 13−protofilament microtubule of about 615 × 8 *nm* = 4.92 *μm* long. Each data point of the coverage *ρ* shown in Fig. 5a has been obtained by averaging over 10^5^ simulated configurations. Histograms shown in Figs 5c,d,g,h,k,l and 7d,e,h, and i have been obtained by computing all the center-to-center distances between nearest neighbors along the protofilament direction (*k* = ∥) averaged over 10^5^ simulated configurations while the snapshots in Fig. 5b,e,f,i,j,m and in Fig. 7c,f,g,j show a zoom in (13 × 40 sites) of a single simulated configuration.

### Numerical solutions

Numerical solutions coverages *ρ*_*p*_ and *ρ*_*h*_ are obtained by using Eqs (2) with (3). See the Supplementary Information, Sec. S2.B.

## Supplementary information

Dynamical decoration of stabilized-microtubules by tau-proteins: supplementary information

## Data Availability

The datasets generated and analysed during the current study are available from the corresponding author on reasonable request.
