# Supplementary material for: Dynamical decoration of stabilized-microtubules by Tau-proteins
Source: Sci Rep. 2019 Aug 28;9:12473. doi: 10.1038/s41598-019-48790-1 (PMC6713733; doi:10.1038/s41598-019-48790-1)
Supplement: Supplementary file 1 — Dynamical decoration of stabilized-microtubules by tau-proteins: supplementary information [file 41598_2019_48790_MOESM1_ESM.pdf]

Dynamical decoration of stabilized-microtubules by tau-proteins: supplementary information

Jordan Hervy[1,2] and Dominique J. Bicout[1,3,*]

[1]Institut Laue-Langevin, 71 Avenue des Martyrs, 38042 Grenoble, France
[2]Laboratory of Physics and Modelling of Condensed Matter, Grenoble Alpes University, CNRS, Grenoble, France
[3]EPSP, TIMC Laboratory, UMR CNRS 5525 Grenoble Alpes University, VetAgro Sup, France

S1. MICROTUBULE COVERAGE

Phase space: In general, analytical solutions of Eq.(2) in the main text providing expressions for ρ_p and ρ_h are not possible because of the non-linearities in the probabilities Φ_p and Φ_h, However, some insights can already be gained by analyzing the phase space (ρ_p, ρ_h) without having solved Eq.(2), i.e., not explicitly determining ρ_p and ρ_h. The relation between ρ_p and ρ_h in the space is given by, equation

$$\frac{\rho_p}{\rho_h} = \kappa\, \frac{(1-\sigma_h\rho_h)^{\sigma_h}}{(1-\sigma_p\rho_p)^{\sigma_p}}\left[1-(1+\sigma_p)\rho_p-(1+\sigma_h)\rho_h\right]^{\sigma_p-\sigma_h} \;,\; \kappa = \frac{k_{\mathrm{eq,p}}}{k_{\mathrm{eq,h}}} \,. \tag{S1}$$

Now, solving Eq.(S1) allows us to portray ρ_h as a function of ρ_p within the triangular section of the phase space. In addition, combining the saturation line and Eq.(S1) allows determining $\rho_{p,s}$ and $\rho_{h,s}$. As analytical solutions of Eq.(S1) are only possible for $\sigma_i = 0, 1$, we consider the solution of Eq.(S1) for $\sigma_p = \sigma_h = 1$,

$$\rho_h(\rho_p,\kappa) = \frac{1}{2}\left[1-\sqrt{1-\frac{4\,\rho_p(1-\rho_p)}{\kappa}}\right] \quad \text{for} \quad \sigma_p=\sigma_h=1 \qquad \text{(see Fig. 4a)}\,. \tag{S2}$$

The position along the trajectory ρ_h *vs* ρ_p depend on the kinetic parameters: the tau:tubulin-dimer ratio, x, and the effective equilibrium constant, $k_{\mathrm{eq}} = k_{\mathrm{eq,p}} + k_{\mathrm{eq,h}}$. For example, in Fig. 4a, the points A and B on the trajectory $\rho_h(\rho_p, \kappa = 1) = \rho_p$ correspond to identical, $k_{\mathrm{eq,p}} = k_{\mathrm{eq,h}} = 1.5$ (i.e., $k_{\mathrm{eq}} = 3$) but to different ratios, $x = 0.15$ and $x = 10$, respectively. This indicates that for a given set of $(k_{\mathrm{eq,p}}, k_{\mathrm{eq,h}})$, the trajectory $\rho_h(\rho_p, \kappa)$ approaches the saturation line with increasing x. Using the saturation line [‡], $1 - 2\rho_{p,s} - 2\rho_{h,s} = 0$ (for $\sigma_p = \sigma_h = 1$), back into Eq.(S1) and solving for $\rho_{p,s}$ and $\rho_{h,s}$ leads to:

$$\rho_{p,s}(\kappa) = \frac{1-\sqrt{1-\kappa\,(1-\kappa)}}{2\,(1-\kappa)}\;, \quad \rho_{h,s}(\kappa) = \frac{\sqrt{1-\kappa\,(1-\kappa)}-\kappa}{2\,(1-\kappa)}\,. \tag{S3}$$

Note that the partial saturation densities $\rho_{p,s}$ and $\rho_{h,s}$ are functions of κ but the total density, $\rho_s = \rho_{p,s} + \rho_{h,s} = 1/2$, is not as it should be.

Diluted regime: The diluted regime, corresponding to a low MT coverage $\rho_p \ll 1$ and $\rho_h \ll 1$, is both especially relevant to the context of axons [39] and interesting as it allows deriving analytical solutions of Eq.(2). In this case, the insertion probabilities Φ's can be linearized to the leading order as,

$$\begin{cases} \Phi_\mathrm{p}(\rho_p,\rho_h) \approx 1-(1+2\sigma_p)\,\rho_p-(1+\sigma_h)\,(1+\sigma_p)\,\rho_h+O(\rho_p\rho_h)\,, \\ \Phi_\mathrm{h}(\rho_p,\rho_h) \approx 1-(1+2\sigma_h)\,\rho_h-(1+\sigma_p)\,(1+\sigma_h)\,\rho_p+O(\rho_p\rho_h)\,. \end{cases} \tag{S4}$$

In addition, the trajectories ρ_h *vs* ρ_p, given in Eq.(S1) can also be linearized as, $\rho_h \simeq \rho_p/\kappa + O(\rho_p{}^2)$. Now, using Eq.(S4) back into Eq.(2) and with linearized phase portraits leads to a system of equations that can be

*Electronic address: bicout@ill.fr

[‡] As the binding in mode "h" is not allowed at the MT seam, the saturation line becomes an approximation. Therefore, for $\sigma_h = 1$ and 13-protofilaments MT, the highest coverage in mode "h" is $\rho_h = 6/13 \approx 0.46$, instead of $1/2$, corresponding to 6 Taus bound in mode "h" (i.e., 12 lattice binding sites are occupied) per MT helix. The correct saturation line is shown in Fig. 4a in dashed line and is given by: $\rho_{h,s}(\rho_{p,s}) = 6/13$ for $0 \leq \rho_{p,s} < 1/26$, and, $\rho_{h,s}(\rho_{p,s}) = 1/2 - \rho_{p,s}$ for $1/26 \leq \rho_{p,s} \leq 1/2$. As it can be seen in Fig. 4a, the saturation line above is exact for $1/26 \leq \rho_{p,s} \leq 1/2$ but becomes an approximation for $\rho_{p,s} < 1/26$ yielding a maximum relative error of about 4%.

solved analytically to give,

$$\begin{cases} \rho_p = \dfrac{1}{2}\left\{\dfrac{\kappa}{1+\kappa}\left(x+\dfrac{1}{\beta_p\, k_{\rm eq,p}}\right)+\dfrac{1}{\beta_p}-\sqrt{\left(\dfrac{\kappa}{1+\kappa}\left(x+\dfrac{1}{\beta_p\, k_{\rm eq,p}}\right)+\dfrac{1}{\beta_p}\right)^2-\dfrac{4\,x\,\kappa}{\beta_p\,(1+\kappa)}}\right\}\ , \\[2ex] \rho_h = \dfrac{1}{2}\left\{\dfrac{1}{1+\kappa}\left(x+\dfrac{1}{\beta_h\, k_{\rm eq,h}}\right)+\dfrac{1}{\beta_h}-\sqrt{\left(\dfrac{1}{1+\kappa}\left(x+\dfrac{1}{\beta_h\, k_{\rm eq,h}}\right)+\dfrac{1}{\beta_h}\right)^2-\dfrac{4\,x}{\beta_h\,(1+\kappa)}}\right\}\ , \end{cases} \tag{S5}$$

where the coefficients β_p and β_h are given by

$$\beta_p = 1+2\sigma_p+\frac{1}{\kappa}\,(1+\sigma_p)\,(1+\sigma_h) \quad \text{and} \quad \beta_h = 1+2\sigma_h+\kappa\,(1+\sigma_p)\,(1+\sigma_h)\ . \tag{S6}$$

For low tau:tubulin-dimer ratio, $x \ll 1$, the coverages become linear functions of x as,

$$\begin{cases} \rho_p \approx x\left(\dfrac{k_{\rm eq,p}}{1+k_{\rm eq,p}+k_{\rm eq,h}}\right) \\[2ex] \rho_h \approx x\left(\dfrac{k_{\rm eq,h}}{1+k_{\rm eq,p}+k_{\rm eq,h}}\right) \end{cases} \quad \Rightarrow \quad \rho \equiv \rho_p+\rho_h \approx x\left(\frac{k_{\rm eff}}{1+k_{\rm eff}}\right)\ . \tag{S7}$$

Interestingly, in the limit $x \ll 1$, the coverage is independent of the binding reaction stoichiometries and, therefore, fitting the experimental data (as, for example, in cosedimentation assays) in this regime would allow determination of the effective equilibrium constant $k_{\rm eff} = k_{\rm eq,h} + k_{\rm eq,p}$. Out of the $x \ll 1$ regime, the ρ's given in Eq.(S5) are non-linear functions of x and depend on stoichiometries and dissociation constants.

S2. METHODS

To make the calculations tractable, the two-dimensional microtubule lattice shown in Fig. S1 is replaced by two coupled linear lattices: protofilament and helix. The underlying approximation is to consider each direction as an independent linear lattice. As illustrated in Fig. S1, for a protofilament "j" (helix "i") , Tau's bound in "p" ("h") mode cover $(1+\sigma_p)$ $[(1+\sigma_h)]$ consecutive sites while those bound in "h" ("p") mode cover 1 site. According to this, the directional coverages $\rho_{\|,p}$ and $\rho_{\|,h}$ can be expressed as a function of ρ_p and ρ_h as,

$$\begin{cases} \rho_{\|,p} = \rho_p \quad \text{and} \quad \rho_{\|,h} = (1+\sigma_h)\,\rho_h\ , \\ \rho_{\perp,h} = \rho_h \quad \text{and} \quad \rho_{\perp,p} = (1+\sigma_p)\,\rho_p\ . \end{cases} \tag{S8}$$

A. Distribution of gaps

The distribution of gaps $f_k(g)$ is defined as the probability of finding a gap of size g (i.e., g consecutive free lattice sites) along the direction k. According to the approximation shown in Fig. S1, two distributions of gaps have been considered: $f_\|(g)$ for the protofilament linear lattice and $f_\perp(g)$ for the helicoidal linear lattice shown in Fig. S1 in red and blue, respectively. McGhee and von Hippel [1] have shown that $f_k(g) \propto {u_k}^g$, where u_k is the conditional probability that a lattice site selected at random along the direction $k =\|, \perp$ is free and that its adjacent site (the immediate up and right site for $k =\|$ and $k =\perp$, respectively) is free as well. Using the normalization condition of the probability for $f_k(g)$, we find:

$$\sum_{g=0}^{g_{\rm m,k}} f_k(g) = 1 \quad \Rightarrow \quad f_k(g) = \left(\frac{1-u_k}{1-u_k^{g_{\rm m,k}+1}}\right){u_k}^g\ , \tag{S9}$$

where $g_{\rm m,k}$ is the maximum size of the physical gap between two bound Tau's along the direction k given by,

$$g_{\rm m,k} = [1-(1+\sigma_p)\,\rho_p-(1+\sigma_h)\,\rho_h] \times \begin{cases} h & \text{for} \quad k =\|\ , \\ p & \text{for} \quad k =\perp\ . \end{cases} \tag{S10}$$

Every microscopic configuration being equally likely, the conditional probability u_k can be obtained as the ratio between the number of free sites, $\#(\text{free sites})_k - 1$, and the total number of objects (free sites plus bound Tau's), $\#(\text{free sites})_k + \#(\text{tau})_k - 1$, forming the lattice along the direction k as,

$$u_k = \frac{\#(\text{free sites})_k - 1}{\#(\text{free sites})_k + \#(\text{tau})_k - 1}\ . \tag{S11}$$

The -1 term in Eq.(S11) accounts for that u_k is zero for a single site. Let ℓ_k (with $\ell_k = h$ and $\ell_k = p$ for $k = \|$ and $k = \perp$, respectively) be the total number of lattice sites along the k direction and, denote by $\rho_{\mathrm{free,k}}$ and ρ_k the proportions of free lattice sites and bound Tau's, respectively, along the direction k, we have:

$$\rho_{\mathrm{free,k}} = 1 - (1+\sigma_p)\,\rho_p - (1+\sigma_h)\,\rho_h\ , \tag{S12}$$

and

$$\rho_k = \begin{cases} \rho_{\|,p} + \rho_{\|,h} \;\equiv\; \rho_p + (1+\sigma_h)\,\rho_h & \text{for} \quad k = \|\ , \\ \rho_{\perp,p} + \rho_{\perp,h} \;\equiv\; \rho_h + (1+\sigma_p)\,\rho_p & \text{for} \quad k = \perp\ . \end{cases} \tag{S13}$$

It follows that, $\#(\text{free sites})_k = \ell_k \rho_{\mathrm{free,k}}$ and $\#(\text{tau})_k = \ell_k \rho_k$, such that,

$$u_k = \frac{\ell_k \rho_{\mathrm{free,k}} - 1}{\ell_k\,(\rho_{\mathrm{free,k}} + \rho_k) - 1} = \begin{cases} \dfrac{1 - (1+\sigma_p)\,\rho_p - (1+\sigma_h)\,\rho_h - 1/h}{1 - \sigma_p\rho_p - 1/h} & \text{for} \quad k = \|\ , \\[2ex] \dfrac{1 - (1+\sigma_p)\,\rho_p - (1+\sigma_h)\,\rho_h - 1/p}{1 - \sigma_h\rho_h - 1/p} & \text{for} \quad k = \perp\ . \end{cases} \tag{S14}$$

In the $h \gg 1$ and $p \gg 1$ limit, the distributions of gaps reads as:

$$f_k(g) \underset{\lim h,p\gg 1}{=} (1-u_k)\; {u_k}^g = \begin{cases} \dfrac{\rho_p + (1+\sigma_h)\,\rho_h}{1-\sigma_p\rho_p}\left[\dfrac{1 - (1+\sigma_p)\,\rho_p - (1+\sigma_h)\,\rho_h}{1-\sigma_p\rho_p}\right]^g & \text{for} \quad k = \|\ , \\[2ex] \dfrac{\rho_h + (1+\sigma_p)\,\rho_p}{1-\sigma_h\rho_h}\left[\dfrac{1 - (1+\sigma_p)\,\rho_p - (1+\sigma_h)\,\rho_h}{1-\sigma_h\rho_h}\right]^g & \text{for} \quad k = \perp\ . \end{cases} \tag{S15}$$

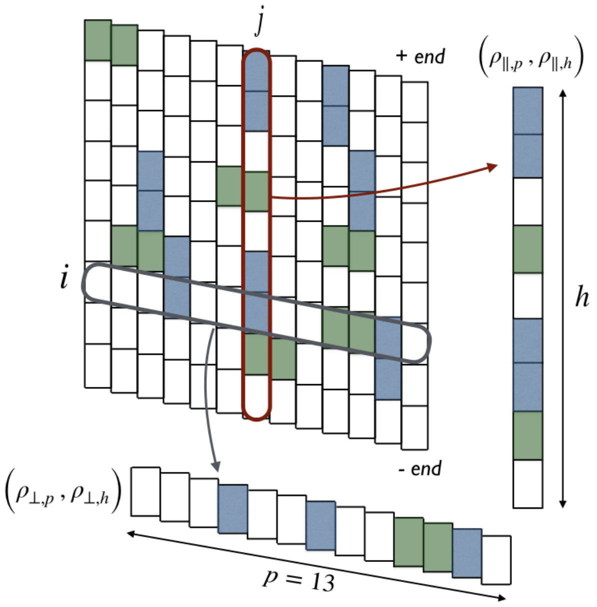

FIG. S1: Illustration of the approximation consisting of replacing the two-dimensional lattice by two linear-lattices: (i) a helix i consisting of $p = 13$ sites with particles bound in "h" mode covering $(1+\sigma_h)$ sites (in green) (coverage, $\rho_{\perp,h}$) and particles bound in "p" mode covering 1 site (in blue) (coverage $\rho_{\perp,p}$) and (ii) a protofilament j consisting of h sites with particles bound in "p" mode covering $(1+\sigma_p)$ sites (in blue) (coverage $\rho_{\|,p}$) and particles bound in "h" mode (in green) covering 1 site (coverage $\rho_{\|,h}$). Adapted from the manuscript of J.H.'s thesis [2].

B. Numerical solutions

Combining of Eqs.(2) and (3) in the main text of the manuscript leads to,

$$\begin{cases} \dfrac{\rho_p}{(x-\rho_p-\rho_h)} = k_{\mathrm{eq,p}} \left[\dfrac{[1-(1+\sigma_p)\rho_p-(1+\sigma_h)\rho_h]^{1+\sigma_p}}{(1-\sigma_p\,\rho_p)^{\sigma_p}} \right], \\ \\ \dfrac{\rho_h}{(x-\rho_p-\rho_h)} = k_{\mathrm{eq,h}} \left[\dfrac{[1-(1+\sigma_h)\rho_h-(1+\sigma_p)\rho_p]^{1+\sigma_h}}{(1-\sigma_h\,\rho_h)^{\sigma_h}} \right]. \end{cases} \tag{S16}$$

For a given set of parameters σ_p, σ_h, $k_{\mathrm{eq,p}}$ and $k_{\mathrm{eq,h}}$, Eq.(S16) is solved for ρ_p and ρ_h using the function *fsolve* in MATLAB for several logarithmically spaced values of x. As shown in Fig. S2, each term in Eq.(S16) corresponds to surfaces in the space (ρ_p, ρ_h) and their intersections leads to an unique point solution of Eq.(S16).

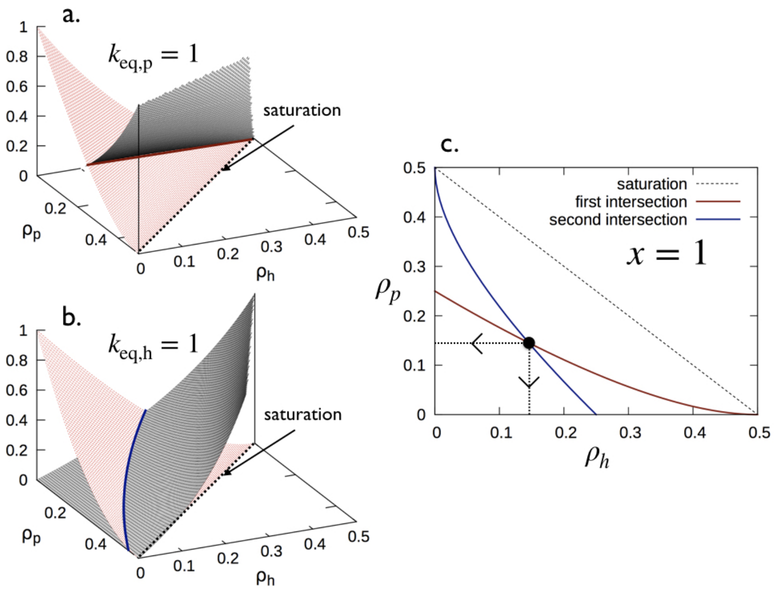

FIG. S2: Graphical representation of the resolution of Eq.(S16) for ρ_p and ρ_h for $\sigma_p = \sigma_h = 1$ with $x = 1$ and $k_{\mathrm{eq,p}} = k_{\mathrm{eq,h}} = 1$. (**a**,**b**) Surface plots corresponding to the first and second lines of Eq.(S16). Dashed lines represent the saturation line given by, $1-(1+\sigma_p)\rho_p-(1+\sigma_h)\rho_h = 0$. (**c**) Intersection between the two lines from Eq.(S16) in (**a**,**b**) leading to an unique point solution of Eq.(S16). Adapted from the manuscript of J.H.'s thesis [2].

---

[1] James D McGhee and Peter H von Hippel. Theoretical aspects of dna-protein interactions: co-operative and non-co-operative binding of large ligands to a one-dimensional homogeneous lattice. *Journal of molecular biology*, 86(2):469–489, 1974.

[2] Jordan Hervy. *Modeling the dynamical interaction Tau Proteins - microtubules.* Theses, Université Grenoble Alpes, November 2018.

S3. BIBLIOGRAPHIC DATA BASE

A. Interaction tau-microtubule

The list of 54 papers below related to the interaction tau-microtubule have been obtained with PubMed and Google Scholar by using the following list of keywords: (i) *Tau microtubule binding site*, (ii) *Tau microtubule interaction*, (iii) *Tau microtubule topology*, (iv) *Tau microtubule binding modes*, (v) *Tau microtubule binding parameters*, (vi) *Tau microtubule binding stoichiometry*, (vii) *Tau microtubule binding dissociation constant*, (viii) *Tau microtubule assembly domain*, (ix) *Tau microtubule decoration*, (x) *Tau binding domain*, (xi) *Tau*

microtubule functions and (xii) *Tau tubulin complex.*

---

[1] M. Ackmann, H. Wiech, and E. Mandelkow. Nonsaturable binding indicates clustering of Tau on the microtubule surface in a paired helical filament-like conformation. *Journal of Biological Chemistry*, 275(39):30335–30343, 2000.

[2] Jawdat Al-Bassam, Rachel S. Ozer, Daniel Safer, Shelley Halpain, and Ronald A. Milligan. MAP2 and tau bind longitudinally along the outer ridges of microtubule protofilaments. *Journal of Cell Biology*, 157(7):1187–1196, 2002.

[3] Jesus Avila, Juan S. Jiménez, Carmen L. Sayas, Marta Bolós, Juan C. Zabala, Germán Rivas, and Felix Hernández. Tau Structures. *Frontiers in Aging Neuroscience*, 8(November):1–10, 2016.

[4] Lidia Bakota, Abdala Ussif, Gunnar Jeserich, and Roland Brandt. Systemic and network functions of the microtubule-associated protein tau: Implications for tau-based therapies. *Molecular and Cellular Neuroscience*, 2016.

[5] Lester I Binder, Anthony Frankfurter, and Lionel I Rebhun. The Distribution of Tau in the Mammalian Central Nervous System.

[6] G. Breuzard, P. Hubert, R. Nouar, T. De Bessa, F. Devred, P. Barbier, J. N. Sturgis, and V. Peyrot. Molecular mechanisms of Tau binding to microtubules and its role in microtubule dynamics in live cells. *Journal of Cell Science*, 126(13):2810–2819, 2013.

[7] K. A. Butner and M. W. Kirschner. Tau protein binds to microtubules through a flexible array of distributed weak sites. *Journal of Cell Biology*, 115(3 I):717–730, 1991.

[8] Miu F. Chau, Monte J. Radeke, Concepción De Inés, Isabel Barasoain, Lori a. Kohlstaedt, and Stuart C. Feinstein. The microtubule-associated protein tau cross-links to two distinct sites on each α and β tubulin monomer via separate domains. *Biochemistry*, 37(51):17692–17703, 1998.

[9] Peter J. Chung, Myung Chul Choi, Herbert P. Miller, H. Eric Feinstein, Uri Raviv, Youli Li, Leslie Wilson, Stuart C. Feinstein, and Cyrus R. Safinya. Direct force measurements reveal that protein Tau confers short-range attractions and isoform-dependent steric stabilization to microtubules. *Proceedings of the National Academy of Sciences*, 112(47):E6416–E6425, 2015.

[10] D O N W Cleveland, Shit-ying Hwo Axd, and Marc Iv. Physical and Chemical Properties of Purified Tau Factor and the Role of Tau in Microtubule Assembly. pages 227–247, 1977.

[11] Don W. Cleveland, Shu Ying Hwo, and Marc W. Kirschner. Purification of tau, a microtubule-associated protein that induces assembly of microtubules from purified tubulin. *Journal of Molecular Biology*, 116(2):207–225, 1977.

[12] Richard L Coffey and Danier L. Purich. Non cooperative binding of the MAP2 binding region to microtubules, 1995.

[13] François Devred, Pascale Barbier, Soazig Douillard, Octavio Monasterio, José Manuel Andreu, and Vincent Peyrot. Tau induces ring and microtubule formation from α β-tubulin dimers under nonassembly conditions. *Biochemistry*, 43(32):10520–10531, 2004.

[14] François Devred, Soazig Douillard, Claudette Briand, and Vincent Peyrot. First tau repeat domain binding to growing and taxol-stabilized microtubules, and serine 262 residue phosphorylation. *FEBS Letters*, 523(1-3):247–251, 2002.

[15] Isabelle L. Di Maïo, Pascale Barbier, Diane Allegro, Cédric Brault, and Vincent Peyrot. Quantitative analysis of tau-microtubule interaction using FRET. *International Journal of Molecular Sciences*, 15(8):14697–14714, 2014.

[16] Aranda R Duan and Holly V Goodson. Taxol-stabilized microtubules promote the formation of filaments from unmodified full-length tau in vitro. *Molecular biology of the cell*, 23(24):4796–4806, 2012.

[17] Aranda R. Duan, Erin M. Jonasson, Emily O. Alberico, Chunlei Li, Jared P. Scripture, Rachel A. Miller, Mark S. Alber, and Holly V. Goodson. Interactions between tau and different conformations of tubulin: Implications for tau function and mechanism. *Journal of Molecular Biology*, 429(9):1424 – 1438, 2017.

[18] Aranda R. Duan, Erin M. Jonasson, Emily O. Alberico, Chunlei Li, Jared P. Scripture, Rachel A. Miller, Mark S. Alber, and Holly V. Goodson. Interactions between Tau and Different Conformations of Tubulin: Implications for Tau Function and Mechanism. *Journal of Molecular Biology*, 429(9):1424–1438, 2017.

[19] Auréliane Elie, Elea Prezel, Christophe Guérin, Eric Denarier, Sacnicte Ramirez-Rios, Laurence Serre, Annie Andrieux, Anne Fourest-Lieuvin, Laurent Blanchoin, and Isabelle Arnal. Tau co-organizes dynamic microtubule and actin networks. *Scientific Reports*, 5(1):9964, 2015.

[20] Caroline Fauquant, Virginie Redeker, Isabelle Landrieu, Jean Michel Wieruszeski, Dries Verdegem, Olivier Laprévote, Guy Lippens, Benoît Gigant, and Marcel Knossow. Systematic identification of tubulin-interacting fragments of the microtubule-associated protein Tau leads to a highly efficient promoter of microtubule assembly. *Journal of Biological Chemistry*, 286(38):33358–33368, 2011.

[21] Benoît Gigant, Isabelle Landrieu, Caroline Fauquant, Pascale Barbier, Isabelle Huvent, Jean Michel Wieruszeski, Marcel Knossow, and Guy Lippens. Mechanism of Tau-promoted microtubule assembly as probed by NMR spectroscopy. *Journal of the American Chemical Society*, 136(36):12615–12623, 2014.

[22] B. L. Goode and S. C. Feinstein. Identification of a novel microtubule binding and assembly domain in the developmentally regulated inter-repeat region of tau. *Journal of Cell Biology*, 124(5):769–781, 1994.

[23] Bruce L. Goode, Miu Chau, Paul E. Denis, and Stuart C. Feinstein. Structural and functional differences between 3-repeat and 4-repeat tau isoforms: Implications for normal tau function and the onset of neurodegenerative disease. *Journal of Biological Chemistry*, 275(49):38182–38189, 2000.

[24] N. Gustke, B. Trinczek, J. Biernat, E. M. Mandelkow, and E. Mandelkow. Domains of τ Protein and Interactions with Microtubules. *Biochemistry*, 33(32):9511–9522, 1994.

[25] N. Gustke, B. Trinczek, J. Biernat, E.-M. Mandelkow, and E. Mandelkow. Domains of tau Protein and Interactions with Microtubules. *Biochemistry*, 33(32):9511–9522, 1994.

[26] N. Hirokawa, Y. Shiomura, and S. Okabe. Tau proteins: the molecular structure and mode of binding on microtubules. *Journal of Cell Biology*, 107(4):1449–1459, 1988.

[27] Ming Hong, Victoria Zhukareva, Vanessa Vogelsberg-ragaglia, Zbigniew Wszolek, Lee Reed, Bruce I Miller, Dan H Geschwind, Thomas D Bird, Daniel Mckeel, Alison Goate, John C Morris, Kirk C Wilhelmsen, Gerard D Schellenberg, John Q Trojanowski, Virginia M Lee, Ming Hong, Victoria Zhukareva, Vanessa Vogelsberg-ragaglia, Zbigniew Wszolek, Lee Reed, Bruce Miller, Dan H Geschwind, Thomas D Bird, Daniel Mckeel, Alison Goate, John C Morris, Kirk C Wilhelmsen, and Gerard D Schellenberg. Mutation-Specific Functional Impairments in Distinct Tau Isoforms of Hereditary FTDP-17. 2017.

[28] Harindranath Kadavath, Romina V. Hofele, Jacek Biernat, Satish Kumar, Katharina Tepper, Henning Urlaub, Eckhard Mandelkow, and Markus Zweckstetter. Tau stabilizes microtubules by binding at the interface between tubulin heterodimers. *Proceedings of the National Academy of Sciences*, 112(24):7501–7506, 2015.

[29] Santwana Kar, Juan Fan, Michael J. Smith, Michel Goedert, and Linda A. Amos. Repeat motifs of tau bind to the insides of microtubules in the absence of taxol. *EMBO Journal*, 22(1):70–77, 2003.

[30] Santwana Kar, Gordon J. Florence, Ian Paterson, and Linda A. Amos. Discodermolide interferes with the binding of tau protein to microtubules. *FEBS Letters*, 539(1-3):34–36, 2003.

[31] Elizabeth H. Kellogg, Nisreen M. A. Hejab, Simon Poepsel, Kenneth H. Downing, Frank DiMaio, and Eva Nogales. Near-atomic model of microtubule-tau interactions. *Science*, 1780(May):eaat1780, 2018.

[32] G Lee, N Cowan, and M Kirschner. The primary structure and heterogeneity of tau protein from mouse brain. *Science*, 239(4837):285–288, 1988.

[33] Gloria Lee, Rachael L. Neve, and Kenneth S. Kosik. The microtubule binding domain of tau protein. *Neuron*, 2(6):1615–1624, 1989.

[34] Xiao Han Li, Jacob A. Culver, and Elizabeth Rhoades. Tau Binds to Multiple Tubulin Dimers with Helical Structure. *Journal of the American Chemical Society*, 137(29):9218–9221, 2015.

[35] R B Maccioni, C I Rivas, and J C Vera. Differential interaction of synthetic peptides from the carboxyl-terminal regulatory domain of tubulin with microtubule-associated proteins. *The EMBO journal*, 7(7):1957–63, 1988.

[36] V. Makrides, M. R. Massie, S. C. Feinstein, and J. Lew. Evidence for two distinct binding sites for tau on microtubules. *Proceedings of the National Academy of Sciences*, 101(17):6746–6751, 2004.

[37] Victoria Makrides, Ting E. Shen, Rajinder Bhatia, Bettye L. Smith, Julian Thimm, Ratneshwar Lal, and Stuart C. Feinstein. Microtubule-dependent oligomerization of tau: Implications for physiological tau function and tauopathies. *Journal of Biological Chemistry*, 278(35):33298–33304, 2003.

[38] E-M Mandelkow, O Schweers, G Drewes, J Biernat, N Gustke, B Trinczek, and E Mandelkow. Structure, Microtubule Interactions, and Phosphorylation of Tau Proteina. *Annals of the New York Academy of Sciences*, 777(1):96–106, 1996.

[39] Eva Maria Mandelkow and Eckhard Mandelkow. Biochemistry and cell biology of Tau protein in neurofibrillary degeneration. *Cold Spring Harbor Perspectives in Biology*, 3(10):1–25, 2011.

[40] Ana M. Melo, Juliana Coraor, Garrett Alpha-Cobb, Shana Elbaum-Garfinkle, Abhinav Nath, and Elizabeth Rhoades. A functional role for intrinsic disorder in the tau-tubulin complex. *Proceedings of the National Academy of Sciences*, 113(50):14336–14341, 2016.

[41] Meaghan Morris, Sumihiro Maeda, Keith Vossel, and Lennart Mucke. The Many Faces of Tau. *Neuron*, 70(3):410–426, 2011.

[42] Marco D. Mukrasch, Martin Von Bergen, Jacek Biernat, Daniela Fischer, Christian Griesinger, Eckhard Mandelkow, and Markus Zweckstetter. The "jaws" of the tau-microtubule interaction. *Journal of Biological Chemistry*, 282(16):12230–12239, 2007.

[43] Efstratios Mylonas, Antje Hascher, Pau Bernado, Eckhard Mandelkow, and Dmitri I Svergun. Domain Conformation of Tau Protein Studied by Solution Small-Angle X-ray Scattering Domain Conformation of Tau Protein Studied by Solution Small-Angle X-ray. 2008.

[44] Hirokawa Nobutaka, Shiomura Yoko, and Okabe Shigeo. Tau Proteins : The Molecular Structure. *The Journal of Cell Biology*, 107(October):1449–1459, 1988.

[45] U Preuss, J Biernat, E M Mandelkow, and E Mandelkow. The 'jaws' model of tau-microtubule interaction examined in CHO cells. *Journal of cell science*, 110 (Pt 6:789–800, 1997.

[46] K. J. Rosenberg, J. L. Ross, H. E. Feinstein, S. C. Feinstein, and J. Israelachvili. Complementary dimerization of microtubule-associated tau protein: Implications for microtubule bundling and tau-mediated pathogenesis. *Proceedings of the National Academy of Sciences*, 105(21):7445–7450, 2008.

[47] Rachel A. Santarella, Georgios Skiniotis, Kenneth N. Goldie, Peter Tittmann, Heinz Gross, Eva Maria Mandelkow, E. Mandelkow, and Andreas Hoenger. Surface-decoration of microtubules by human tau. *Journal of Molecular Biology*, 339(3):539–553, 2004.

[48] Iwan A T Schaap, Bernd Hoffmann, Carolina Carrasco, Rudolf Merkel, and Christoph F. Schmidt. Tau protein binding forms a 1 nm thick layer along protofilaments without affecting the radial elasticity of microtubules. *Journal of Structural Biology*, 158(3):282–292, 2007.

[49] O Schweers, E Schönbrunn-Hanebeck, a Marx, and E Mandelkow. Structural studies of tau protein and Alzheimer paired helical filaments show no evidence for beta-structure. *The Journal of biological chemistry*, 269(39):24290–7, 1994.

[50] C. W. Scott, A. B. Klika, M. M.S. Lo, T. E. Norris, and C. B. Caputo. Tau protein induces bundling of microtubules in vitro: Comparison of different tau isoforms and a tau protein fragment. *Journal of Neuroscience Research*, 33(1):19–29, 1992.

[51] Nicolas Sergeant, André Delacourte, and Luc Buée. Tau protein as a differential biomarker of tauopathies. *Biochimica et Biophysica Acta - Molecular Basis of Disease*, 1739(2):179–197, 2005.

[52] Alain Sillen, Pascale Barbier, Isabelle Landrieu, Sylvie Lefebvre, Jean-michel Wieruszeski, Arnaud Leroy, Vincent Peyrot, and Guy Lippens. NMR Investigation of the Interaction between the Neuronal Protein Tau. pages 3055–3064,

2007.
[53] Philipp O. Tsvetkov, Alexander A. Makarov, Soazig Malesinski, Vincent Peyrot, and Francois Devred. New insights into tau-microtubules interaction revealed by isothermal titration calorimetry. *Biochimie*, 94(3):916–919, 2012.
[54] Holger Wiue, Gerard Drewes, Jacek Biernat, Eva-Maria Mandelkow, and Eckhard Mandelkow. Alzheimer-like Paired Helical Filaments and Antiparallel Dimers Formed from Microtubule-associated Protein Tau In Vitro. 118(3):573–584, 2000.

B. Structure of the microtubule lattice

The list of 16 papers below related to the structure of the microtubule lattice have obtained with PubMed and Google Scholar by using the following list of keywords: (i) *Microtubule structure*, (ii) *Microtubule lattice*, (iii) *Microtubule surface lattice* and (iv) *Microtubule lattice seam.*

---

[1] Anna Akhmanova and Michel O. Steinmetz. Tracking the ends: a dynamic protein network controls the fate of microtubule tips. *Nature Reviews Molecular Cell Biology*, 9(4):309322, 2008.
[2] Linda A. Amos. Microtubule structure and its stabilisation. *Organic & Biomolecular Chemistry*, 2(15):2153, 2004.
[3] D. Chretien, F. Metoz, F. Verde, E. Karsenti, and R. H. Wade. Lattice defects in microtubules: Protofilament numbers vary within individual microtubules. *Journal of Cell Biology*, 117(5):10311040, 1992.
[4] Denis Chretien and Stephen D Fuller. Microtubules switch occasionally into unfavorable configurations during elongation. *Journal of Molecular Biology*, 298(4):663676, 2000.
[5] Denis Chretien and Richard H. Wade. New data on the microtubule surface lattice. *Biology of the cell / under the auspices of the European Cell Biology Organization*, 71(1-2):161174, 1991.
[6] Kenneth H. Downing and Eva Nogales. Tubulin and microtubule structure, 1998.
[7] Viktoria Hunyadi, Denis Chretien, Henrik Flyvbjerg, and Imre M. Janosi. Why is the microtubule lattice helical? *Biology of the Cell*, 99(2):117128, 2007.
[8] Viktoria Hunyadi, Denis Chretien, and Imre M. Janosi. Mechanical stress induced mechanism of microtubule catastrophes. *Journal of Molecular Biology*, 348(4):927938, 2005.
[9] A. A. Hyman, D. Chretien, I. Arnal, and R. H. Wade. Structural changes accompanying GTP hydrolysis in microtubules: Information from a slowly hydrolyzable analogue guanylyl-methylene-diphosphonate. *Journal of Cell Biology*, 128(1-2):117125, 1995.
[10] Masahide Kikkawa, Takashi Ishikawa, Takao Nakata, Takeyuki Wakabayashi, and Nobutaka Hirokawa. Direct visualization of the microtubule lattice seam both in vitro and in vivo. *Journal of Cell Biology*, 127(6 II):19651971,
[11] Huilin Li, David J. DeRosier, William V. Nicholson, Eva Nogales, and Kenneth H. Downing. Microtubule structure at $8\mathring{A}$ resolution. *Structure*, 10(10):13171328, 2002. 1994.
[12] Eckhard Mandelkow and Eva Maria Mandelkow. Microtubule structure. *Current Opinion in Structural Biology*, 4(2):171179, 1994.
[13] P. Meurer-Grob, J. Kasparian, and R. H. Wade. Microtubule structure at improved resolution. *Biochemistry*, 40(27):80008008, 2001.
[14] Eva Nogales, Michael Whittaker, Ronald A. Milligan, and Kenneth H. Downing. High-resolution model of the microtubule. *Cell*, 96(1):7988, 1999.
[15] David Sept, Nathan A. Baker, and J. Andrew McCammon. The physical basis of microtubule structure and stability. *Protein Science*, 12(10):22572261, 2009.
[16] Lewis G Tilney, Joseph Bryan, and Doris J Bush. Microtubules evidence for 13 protofilaments. *The Journal of Cell Biology*, 59:267275,1973.
